# A Genomic Survey of *Mayetiola destructor* Mobilome Provides New Insights into the Evolutionary History of Transposable Elements in the Cecidomyiid Midges

**DOI:** 10.1371/journal.pone.0257996

**Published:** 2021-10-11

**Authors:** Wiem Ben Amara, Hadi Quesneville, Maha Mezghani Khemakhem

**Affiliations:** 1 Laboratory of Biochemistry and Biotechnology (LR01ES05), Faculty of Sciences of Tunis, University of Tunis El Manar, Tunis, Tunisia; 2 INRAE, URGI, Université Paris-Saclay, Versailles, France; 3 INRAE, BioinfOmics, Plant Bioinformatics Facility, Université Paris-Saclay, Versailles, France; University of Bari: Universita degli Studi di Bari Aldo Moro, ITALY

## Abstract

The availability of the Whole-Genome Sequence of the wheat pest *Mayetiola destructor* offers the opportunity to investigate the Transposable Elements (TEs) content and their relationship with the genes involved in the insect virulence. In this study, de novo annotation carried out using REPET pipeline showed that TEs occupy approximately 16% of the genome and are represented by 1038 lineages. Class II elements were the most frequent and most TEs were inactive due to the deletions they have accumulated. The analyses of TEs ages revealed a first burst at 20% of divergence from present that mobilized many TE families including mostly *Tc1/mariner* and *Gypsy* superfamilies and a second burst at 2% of divergence, which involved mainly the class II elements suggesting new TEs invasions. Additionally, 86 TEs insertions involving recently transposed elements were identified. Among them, several MITEs and *Gypsy* retrotransposons were inserted in the vicinity of SSGP and chemosensory genes. The findings represent a valuable resource for more in-depth investigation of the TE impact onto *M*. *destructor* genome and their possible influence on the expression of the virulence and chemosensory genes and consequently the behavior of this pest towards its host plants.

## Introduction

Transposable elements (TEs) are repeated and dispersed DNA sequences able to move from one locus to another in the same or among different chromosomes of the same host [1]. They represent an important fraction of eukaryote genomes that they colonize, and they are key players in the genome evolution and diversity [2, 3].

TEs are classified according to their mechanism of transposition into two main classes [4]. Class I elements, or retrotransposons, require an RNA intermediate and transpose by the “copy-paste” model; while Class II elements, termed DNA transposons, move through a DNA intermediate and ensure transposition by the “cut-paste” model.

The TE content varies significantly between different organisms [5]. In insects, for example, it ranges from 1% in the Antarctic midge genome to 65% in the migratory locust [6, 7]. Due to their mobility and dissemination in the genome, TEs are thought to have a considerable contribution to the plasticity and dynamics of the host’s genome as well to the evolution of its architecture. Indeed, TEs lead to chromosomal rearrangements like deletions, duplications, inversions, and translocations through ectopic recombination such as the chromosomal inversions caused by Foldback TEs in *Drosophila buzzatii* [8, 9]. TEs can also be considered as innovator sequences taking new genetic information to the genome, notably promoters and splicing sites. Different examples of gene expression under the control of TEs have been reported in different species, and a catalogue of genes affected by TEs (C-GATE) has been established [10–12]. These changes induced by mobile elements are not always deleterious; some TEs can be drivers of genomic innovation and are recruited as new genes for different biological functionalities conferring selective advantages to the host [13–15]. Hence, some TE insertions play an important role in the acquisition of insecticide resistance [16], variation in the courtship songs for partnership [17] and climate adaptation [18]. These examples of various evolutionary implications of TEs and the availability of whole genome sequences (WGS) have impacted research on insects. Indeed, in the last two decades, the development of bioinformatics tools has enabled TEs characterization of several insect genomes and different tools for TE annotation have been developed and made available. These tools include de novo methods such as REPET, RepeatModeler and PiRATE pipelines [19–21], knowledge-based TE detection methods like Repeatmasker and TEseeker [22, 23] as well as comparative population genomics methods using PoPoolationTE2 [24]. These various tools have hastened insights concerning the molecular genetic basis of many insect phenotypes and recent findings indicate that TEs are involved in insect adaptations and are powerful facilitators of their genome evolution [25–28].

Among insects, *D*. *melanogaster*, has been the primary research organism for evolutionary studies focusing on TEs [29]. However, this species only cannot serve as a model for all of the insect diversity; it is clearly not suitable for studying several interesting phenotypes found among insects, such as virulence to plant resistance. The Hessian fly *Mayetiola destructor* (Say, 1817) (Diptera, Cecidomyiidae) is one of the most economically devastating pests damaging wheat and barley cultures all over the world [30–33]. In Georgia, roughly $20 million in losses from Hessian fly damage were reported [34]. In Tunisia, it was detected in 60.33% and 51.5% of all sampled durum and bread wheat fields [35]. In Morocco, losses were estimated at around 42% on bread wheat and 32% on durum wheat as reported by [36]. The Hessian fly is considered as a genetically tractable animal model to study the insect-plant interactions [37, 38]. In fact, *M*. *destructor*, was the first insect shown to have a gene-for-gene interaction with its host plant [39] and for many years, it was the only well documented model used for studying the gene-for-gene relationship in plant-insect interactions [37]. This interaction involves the plant genome containing a large repertoire of resistance genes *R* and the insect avirulence genes (*Avr)* as well as multiple virulence gene families like the Secreted Salivary Gland Proteins (SSGP), which facilitate the insect’s adaptation to its host defense [37, 40]. To better understand the genetic interaction between Hessian fly and its host, several studies have focused on the identification of transposable elements and specifically the mariner family [41–43] as well as their insertion sites polymorphisms [44]. The genome of *M*. *destructor* was sequenced in 2010 to represent the first reference cecidomyiid Whole-Genome Sequence (WGS), nevertheless, it is still underexplored, and the search and characterization of other TEs will allow a better understanding of TEs evolution and *Mayetiola* adaptation.

In the current work, we conducted a genome-wide analysis of the Hessian fly mobilome using *in silico* approaches. The aim of this study was to provide an accurate description of TEs and to explore genes in their proximity. This study will deepen our knowledge in TEs dynamics, which is fundamental in understanding the genome evolution and adaptation in this cecidomyiid fly.

## Materials and methods

The *Mayetiola destructor* genome sequence (*Mdes1*.*0* version) available at NCBI under accession PRJNA45867 was used for TEs annotation. This genome of 153 Mb was assembled in 36404 contigs and 24503 scaffolds with a N50 of 14 kb and 756 kb, respectively. About 59% of the genome sequence was anchored to the four chromosomes of *M*. *destructor* [45].

### TE de novo detection and annotation

The annotation of *M*. *destructor* TEs was carried out using the two pipelines TEdenovo and TEannot of the REPET package v2.3 with default parameters (PMID: 24786468, PMID: 21304975, https://urgi.versailles.inra.fr/Tools/REPET). The TEdenovo pipeline was used first to detect TEs in *M*. *destructor* genome [20]. To begin, the genome was cut into batches which were then compared to themselves by “all against all” method using BLASTER [46]. Second, the detected repetitive HSPs (High Scoring Pairs) were clustered into TE families using GROUPER [46], RECON [47], and PILER [48] tools.

For each identified TE family, copies were aligned to allow the generation of consensuses which were then classified according to their structural features (LTR, TIR, polyA tail, ORF…) as well as their similarities with reference TEs from Repbase updated database v19.12 [49], Pfam databases [50] and HMM profiles. Classification was made by PASTEC tool [51] following the classification system described by Wicker, Sabot [52].

The TEannot pipeline, including BLASTER [46], REPEATMASKER 3.2.6 [53] and CENSOR [49] tools, was used to annotate TE copies using as input the identified consensuses library generated by a TE *de novo* pipeline. TEannot pipeline was launched three times. The first turn was used to annotate all TE sequences. The second was performed using consensuses that encompass at least one full-length copy (called FLC, *i*.*e*. copies that covers more than 95% of the corresponding consensus) in order to improve the TE annotation quality of the sequences [54]. Subsequently, a third turn was launched using as TE library these filtered consensuses, but removing also consensuses classified as SSRs, and noCats generated from less than 10 copies to estimate *M*. *destructor* TE coverage. All the TE consensus sequences were also manually curated to characterize their structural features and to filter them from the artefactual chimeras and duplicates.

To characterize MITE sequences, the Hessian fly genome assembly was submitted to the MITE Tracker tool [55] and putative MITEs were aligned and clustered into families by Vsearch [56] based on target sites duplication (TSD) and Terminal Inverted Repeat (TIR) sequences.

### Phylogenetic analyses

The classification of TEs consensuses identified in *M*. *destructor* was inferred per superfamily using reference elements from GenBank and Repbase [57, 58]. The trees were constructed using the Maximum Likelihood method following the HKY85 model [59] which was arbitrarily chosen. Phylogenetic trees were built with a bootstrap analysis of 1000 replicates using the MEGA 6 program [60] and displayed with iTOL v3 program [61].

### TE sequences age estimates

TEs divergences and ages were estimated using the terminal branch forks lengths of TE copy phylogenies. High identity scores refer to few accumulated mutations since their divergence from their ancestor and are consequently recent [62, 63].

Copies of each TE family that are more than 100 bp in length were aligned using refalign from the REPET package implementing the Needleman-Wunsch algorithm [64] for pairwise sequence alignment with the consensus sequence considered as reference. A master-slave multiple alignment was then obtained by stacking these pairwise alignments.

Trees were then inferred for each TE family using the PhyML program [65, 66] based on Maximum Likelihood method with the HKY85 model [59].

Pairs of aligned copies deriving from the same terminal branch forks were then selected to estimate their nucleic divergence. Nucleotide substitution per base pair count between closest copies pairs might allow the estimation of the age of the most recent transposition events. Therefore, the number of differences between these sequences corresponds to the substitutions that occurred after the sequence duplication. TE copy age was expressed in substitution number per base pair [62].

### TEs insertion sites

To study TEs flanking regions and search for possible insertions near genes, we used bedtools-closest [67, 68] by combining TEs Generic File Format (GFF) annotations from TEannot with those of the Official Gene Set (OGS) obtained by automatic annotations by the MAKER2 program (*M*. *destructor* 20163 genes are available at i5k Workspace@NAL database) [45, 69]. This program finds the nearest features to TE copies and/or overlapping ones in the genome. TEs insertions were then visualized by Webapollo [70] and Integrative Genomics Viewer IGV 2.3 [71, 72]. The insertions were finally classified according to the TE insertion sites in the genes, their orientations, and the distance between genes and TEs.

## Results

### TEs in the Hessian fly genome

The screening of TEs in the genome of *M*. *destructor* with REPET pipelines allowed us to identify 1168 consensuses from which 1038 lineages containing at least one full length copy (FLC) were extracted. About 16.84% of the genome of *M*. *destructor* was found to be composed of repetitive sequences including TEs (Table 1).

**Table 1:** Global statistics of TEs identified in the *Mayetiola destructor* genome.

			First round of REPET (all the copies)			Second round of REPET (FLC)		
Code	Order		Number of consensus	Genome coverage (%)	Genome coverage (bp)	Consensus number	Genome coverage (%)	Genome coverage (bp)
RLX	LTR		45	**1.26**	1932824	38	**1.17**	1789590
RIX	Non- LTR	LINE	28	**0.37**	568834	28	**0.39**	590859
RSX		SINE	3	**0.04**	65866	3	**0.03**	52834
RPX	*Penelope*		5	**0.05**	72120	2	**0.02**	26689
RXX	Retrotransposons TRIM		3	**0.01**	13707	2	**0.01**	12372
**Total Class I**			**84**	**1.73**	**2653351**	**73**	**1.62**	**2472344**
DTX	TIR		90	**1.13**	1727136	79	**1.03**	1575291
	MITEs		83	**0.89**	1360651	84	**0.87**	1338036
DHX	*Helitron*		7	**0.08**	120684	7	**0.08**	121020
DMX	*Maverick*		11	**0.15**	228938	10	**0.15**	228968
DXX	Unknown transposons		1	**0.01**	19151	1	**0.01**	19148
**Total Class II**			**192**	**2.26**	**3456560**	**181**	**2.14**	**3282463**
PHG	-		44	**0.98**	1507976	43	**0.981**	1502303
noCat	-		843	**11.85**	18148362	738	**11.6**	17775484
SSR	-		5	**0.02**	31970	3	**0.02**	32341
Total	-		**1168**	**16.84**	**25798219**	**1038**	**16.36**	**25064944**

**FLC**: Full Length Copies; **TRIM**: Terminal-repeat transposon in miniature; **PHG**: Potential Host Genes; **noCat**: Unknown repetitions; **DHX**: *Helitrons*; **DMX**: *Maverick*; **DTX**: TIRs elements; **DXX**: Other Class II transposons; **RIX**: LINEs; **RLX**: LTR retrotransposons; **RSX**: SINEs; **RXX_TRIM**: Terminal Repeat Transposons in Miniature; **RPX**: *Penelopes*.

The annotation using the 1038 FLC families showed 16.36% repeats genomic fraction, presenting a slight difference with that of the first TEannot run. By removing consensuses without FLC copies, consensus sequences corresponding to satellites and poorly supported consensus with no TE hallmark, we finally end up with 350 TE consensuses covering 9.39% of the genome sequence.

**Fig 1:**
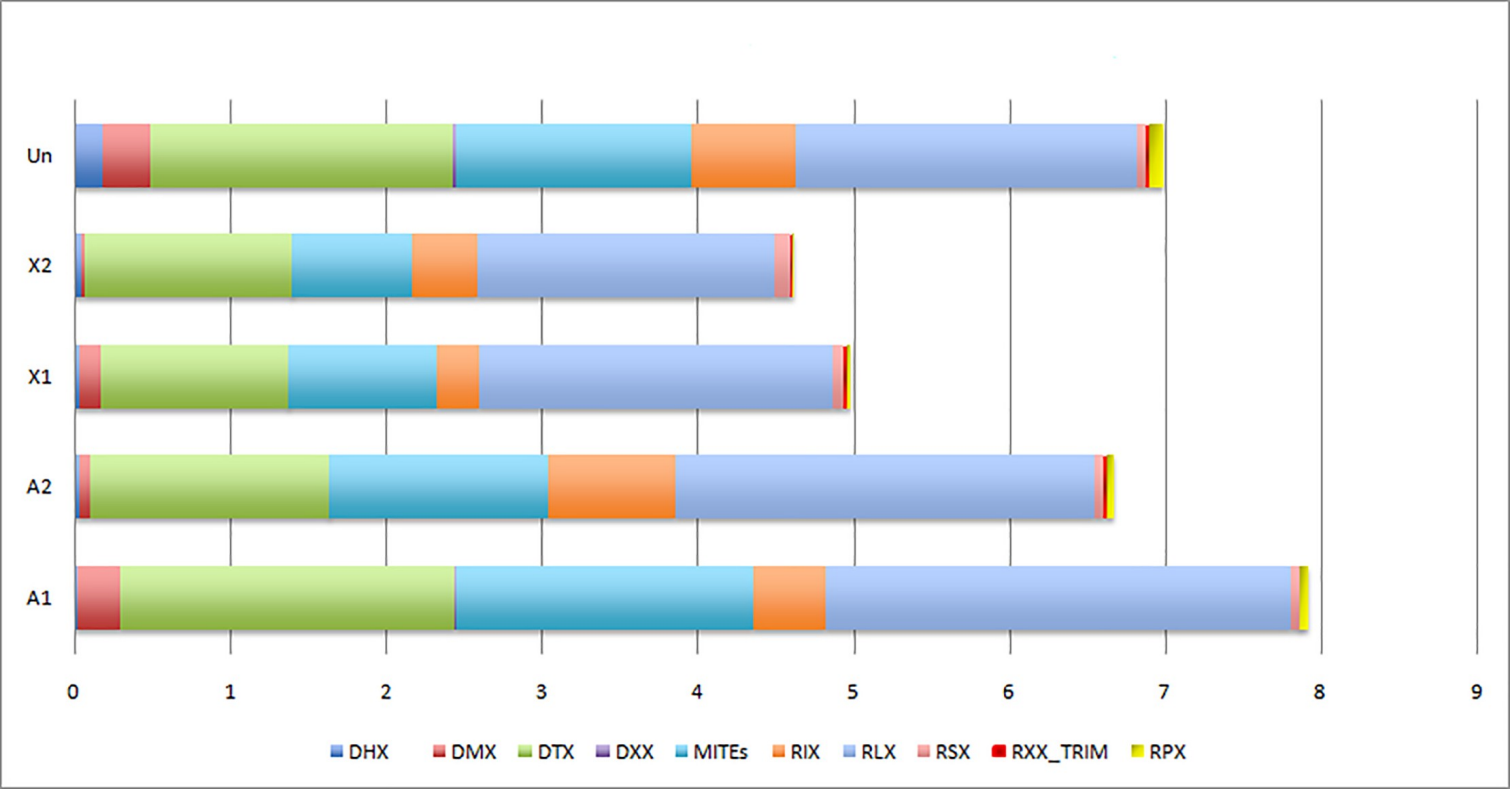
Proportions of TEs in *Mayetiola destructor* chromosomes. A1 and A2: autosomes. X1 and X2: heterosomes. Un: scaffolds non-anchored to chromosomes (unplaced) are considered as a fifth chromosome. DHX: *Helitrons*. DMX: *Maverick*. DTX: TIRs elements. DXX: Other Class II transposons. RIX: LINEs. RLX: LTR retrotransposons. RSX: SINEs. RXX_TRIM: Terminal Repeat Transposons in Miniature. RPX: *Penelopes*. The x-axis refers to the percentage of TEs per chromosomes.

The consensuses were classified according to their structural features into at least five and four orders from TE Class I and Class II, respectively. Interestingly, this TE annotation showed in all the cases a higher frequency of Class II elements than Class I elements covering respectively 2.26 and 1.73% of the genome. Class I includes LTR elements covering 1.17% of the genome followed by the LINEs (0.39%), the SINEs (0.03%) then the Penelope elements (0.02%) as well as the Terminal-repeat Retrotransposon In Miniature (TRIM) (0.01%), while Class II includes TIR elements (1.03%), Miniature Inverted Terminal-repeat Transposable Element (MITEs) (0.87%) followed by the *Maverick* and *Helitron* elements covering respectively 0.15 and 0.08% of the insect genome (Table 1). However, many consensuses, generated from repeats identified by similarity between genomic scaffolds, could not be classified and did not show any obvious similarities with reference TEs from Repbase or with Pfam and HMM profiles and then were considered as noCats. The distribution of TEs per chromosomes revealed that all TE orders were present among the four *M*. *destructor*’s chromosomes and the unplaced scaffolds (considered as the fifth chromosome) with the dominance of the LTR and TIR elements followed by MITEs and LINEs in all the chromosomes (Fig 1, S1 Table).

#### Class I elements

**Fig 2:**
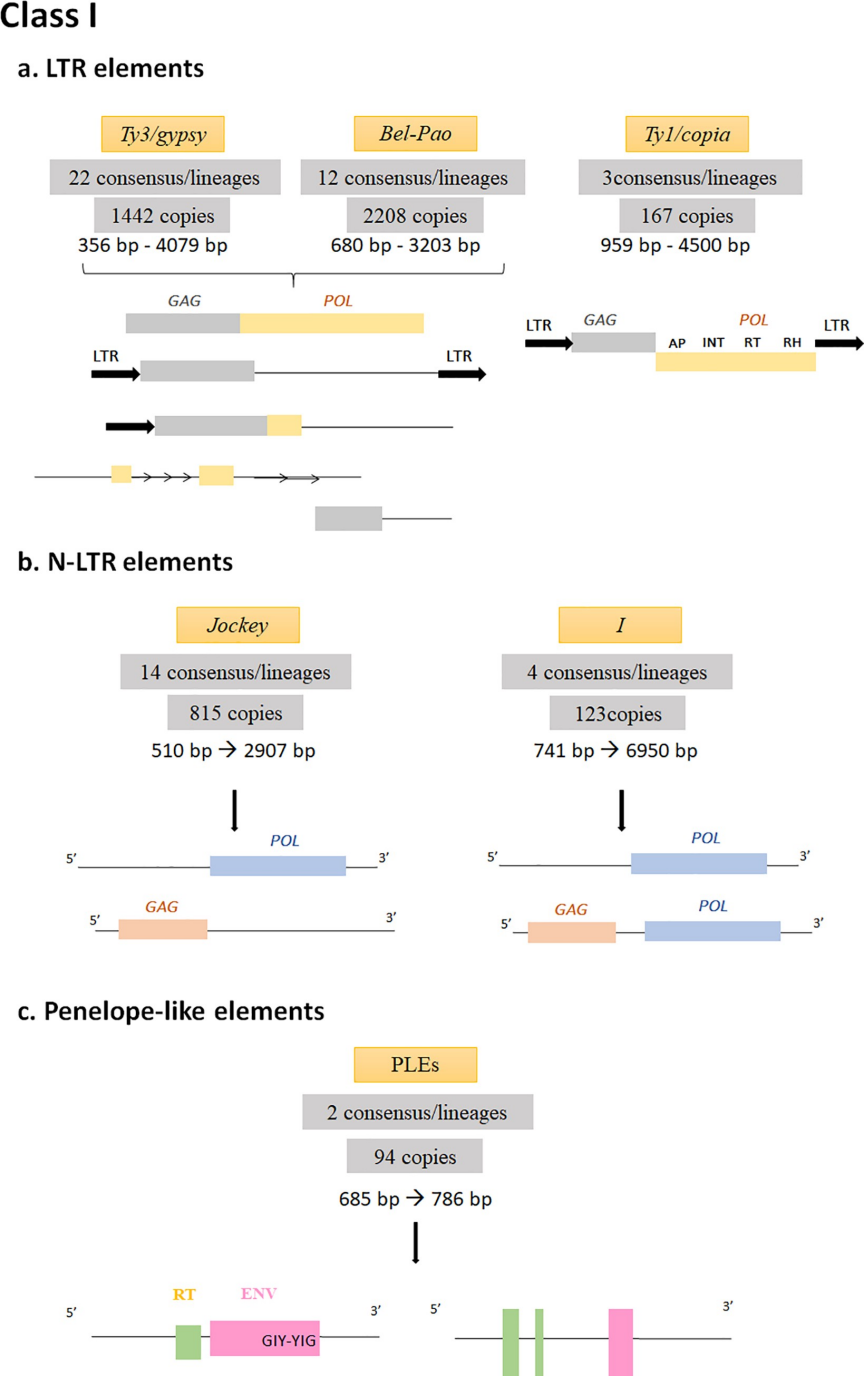
Schematic representation of Class I TE structures in *Mayetiola destructor* genome. The numbers of copies and consensuses representing lineages of each Class I order are mentioned. Full length copies and some inactive forms of Class I elements are mentioned **a.** LTR elements with the three identified superfamilies *Ty3/gypsy*, *Bel-Pao* and *Ty1/copia*. LTR are indicated by black bold arrows, *gag* and *pol* domains are respectively represented in purple and orange rectangles with gene names written in bold letters, deletions are represented by thin black lines, repeated motifs are indicated in thin repeated arrows. **b.** N-LTR elements with the two identified superfamilies *Jockey* and *I* elements. *Gag* and *pol* domains are respectively represented by pink and blue rectangles, deletions are indicated in thin black lines. **c.** *Penelope*-like elements. *RT* and *Env* domains are shown respectively in green and pink rectangles. GIY-YIG motif is indicated as well.

REPET annotation showed that *M*. *destructor* genome has a TE Class I coverage of about 2.5Mb including LTR, non-LTR and TRIMs covered by 73 consensuses (S2 Table). Three LTR superfamilies were identified according to their structural features of reverse transcriptase and *gag-pol* domains: *Bel-Pao* presenting 43% of the Class I elements followed by *Ty3/gypsy* (32%) then *Ty1/copia* presenting 5% of Class I elements. *Bel-Pao* elements belong to only 12 different lineages whose median lengths range from 680 to 3203 bp with truncated *gag-pol* domains while *Ty3/gypsy* are more diverse and belong to 22 lineages whose lengths range from 356 to 4079 bp (S2 Table). All *Ty3/gypsy* copies seem to be inactive due to deletions covering one or both domains *gag-pol* as well as tandem repeats occurring in some of the rudiment copies (Fig 2). In *Ty2/copia*, only one copy has all the domains involved in the LTR transposition process and could be consequently potentially active (Fig 2). This copy showed a conserved target site duplication and displayed 90% of LTR identity.

Concerning TRIM elements, they include 45 copies, which belong to two lineages whose consensus size about 350 bp with LTRs ranging from 100 bp to 190 bp flanking non-coding regions that ranged from 10 to 150 bp.

Two non-LTR superfamilies were identified according to the similarities with the Reverse Transcriptase *RVT_1_RT* domains and the *exo-end-phosphatase* as well as the LINE references available in Repbase: *Jockey* and *I* superfamilies presenting respectively 15% and 2% of the Class I elements.

As for the *Jockey* elements, they belong to 14 lineages and their median lengths vary from 510 to 2907 bp due to the deletions that occurred in different regions. Only one consensus had a normal ORF1 while all the others had only their ORF2 of *pol* (Fig 2). Nevertheless, some of the deleted copies were inserted next to non-sequenced regions (gaps) which could contain 5’ region.

Concerning Class I elements, the distribution was into four lineages with median lengths varying from 741 to 6950 bp. Except for one *I* copy of about 6900 bp, which accounts for all the domains *gag-pol* necessary for transposition process, all the remaining copies have only the ORF2 of *pol* gene (Fig 2). Like the Jockey elements, some of the copies were located in the vicinity of gaps in *M*. *destructor*’s genome, which could explain the absence of 5’ region.

#### Class II elements

The annotation of Class II transposons in the genome of *M*. *destructor* allowed the identification of 181 consensuses representing different transposons lineages. These consensuses enabled the annotation of 3.3 Mb covered by Class II transposons (S2 Table).

*TIR elements*. The identified TIR elements represent 68% of the Class II elements annotated in the genome of *M*. *destructor* and include five superfamilies, which are the *Tc1/mariner*, *hAT*, *Mutator*, *CACTA* and *Pif-Harbinger*. MITE*s* are the most dispersed and frequent TIRs covering 1.3 Mb by copies belonging to 84 families.

- ***Tc1/mariner* elements**

**Fig 3:**
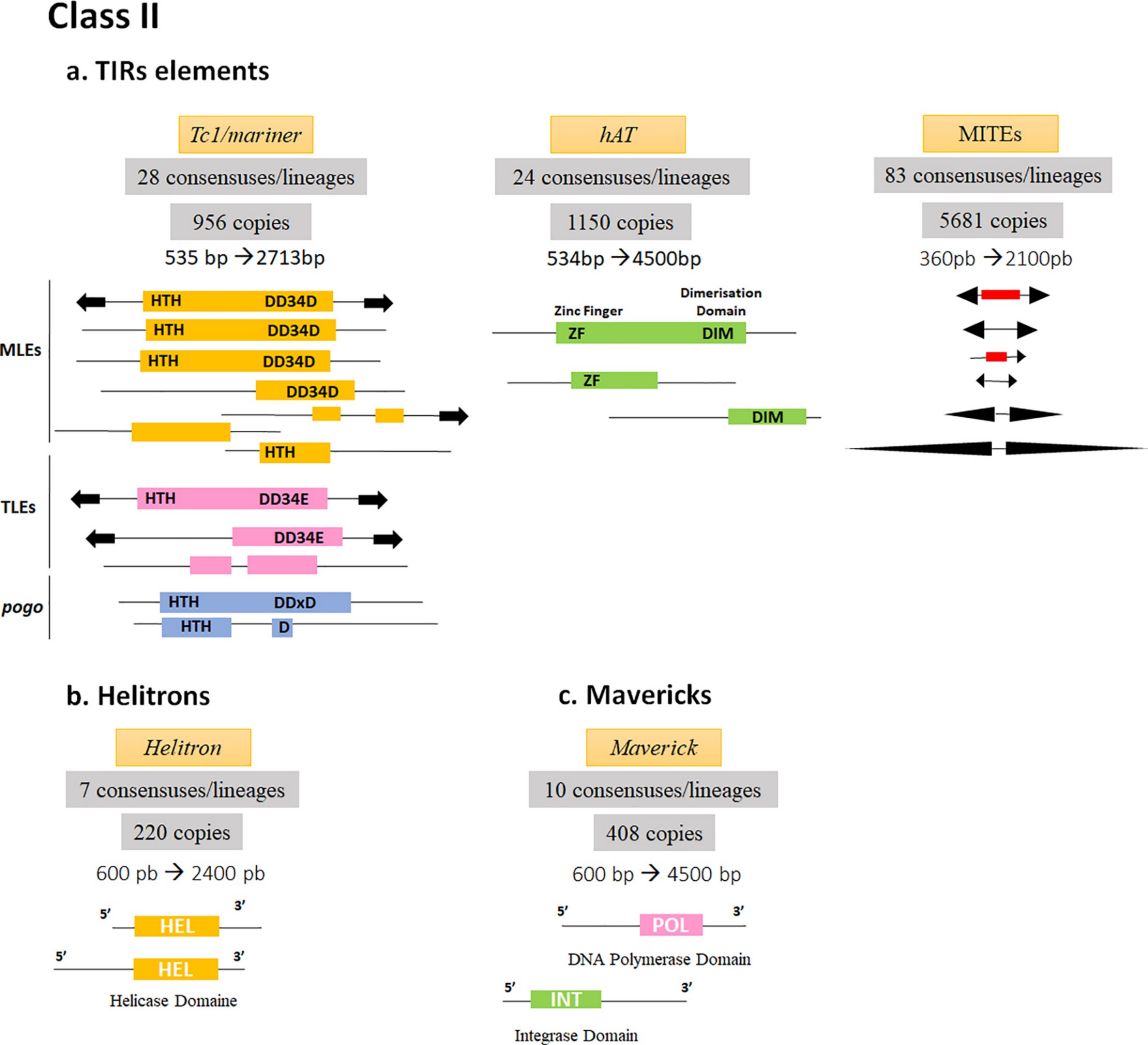
Schematic representation of class II TE structures in *Mayetiola destructor* genome. The numbers of copies and consensuses representing lineages of each Class II order are mentioned. Full-length copies and some inactive forms of DNA transposons are mentioned **a.** TIR elements. Only the most frequent superfamilies were represented, they correspond to the *mariner*-like elements (MLEs) and *hAT* as well as inactive TIR forms, the MITEs. TIR structures are represented by black bold arrows and ORFs are indicated by rectangles. Internal deletions are represented by thin black lines. In *Tc1/mariner* like elements, the Helix Turn Helix (HTH) region of the DNA binding domain is indicated as well as the three aspartic or glutamic amino acids (DDxD/E) characterizing the C-ter Tc1/mariner-like elements. In *hAT* transposons, the Zinc Finger and Dimerisation domains are shown in bold letters. **b**. and **c.** The inactive forms of *helitrons* and *Mavericks* are represented showing the 5’ or the 3’ regions with their appropriate domains.

It is the most diverse superfamily in the genome of *M*. *destructor* showing 32% of the Class II transposon including 956 copies, which belong to 28 different lineages, and cover 347209 bp (S2 Table). Most copies were fragmented due to deletions that occurred in different parts of the TEs (Fig 3), while only 6.88% of the annotated *Tc1/mariner* showed sizes bigger than 900 bp including four potentially active copies with conserved target site duplication (TSD).

Analyses of consensus sequences of this superfamily have shown their distribution into three major families: The *Mariner*-like Elements (MLEs), the *Tc1*-Like-Elements (TLEs) and the *pogo* elements. The MLEs are the most diverse in subfamilies with 21 lineages from which different whole copies were potentially active. The latter have a complete ORF and their transposases contain the HTH and DDD domains necessary for the *MLE* transposition (Fig 3). However, the TLEs and the *pogo* elements were less diverse and less frequent and distributed respectively into three and four groups. No complete copies have been detected in these two families. Indel mutations occur in their DDD/E catalytic domains in the C terminal region (C-ter) and the N terminal HTH domain (N-ter).

- ***hAT* elements**

The *hAT* elements constitute the second major superfamily in the Class II elements of *M*. *destructor* covering 30% of identified Class II transposons. Analyses have shown that 1150 *hAT* insertions belonged to 24 lineages with a genome coverage of 325844 bp (S2 Table). Notably, 78% of those copies were found to have sizes less than 1000 bp originating from deletions involving the N-ter or the C-ter as well as the TIR sequences. However, other copies were deleted because of the gaps between the contigs; indeed, several *hAT* copies were localized in the contigs or scaffolds ends. About ten copies whose sizes ranged from 2 kb to 4.5 kb contain the potential domains necessary for *hAT* transposition. Nevertheless, terminal inverted repeats could not be identified. Moreover, only two *hAT* consensuses were highly similar (85% of identity), while all others (representing the different lineages) showed a high diversity with identity percentage ranging from 34 to 56%.

- ***Mutator* elements**

*Mutator-like elements* (MULEs) represent only 2% of the Class II transposons identified in the genome of *M*. *destructor* (Fig 3). Twenty-eight copies have been annotated by two consensuses covering 20893 bp of the genome of this pest. The identified MULE copies were small sized as the deletions cover the TIRs and some regions of their ORFs.

- ***CACTA* elements**

These elements cover 29,431 bp of *M*. *destructor* genome and constitute 3% of identified Class II elements. They belong to two lineages whose consensuses are 1042 and 1664 bp of length, respectively. One of the *CACTA* copies showed TIRs of 334 bp containing in their extremities the *CACTA* motif modified into C**T**C**G**A. They are much longer than known *CACTA* TIRs whose sizes are of 13 bp to 30 bp.

- ***Pif-harbinger* elements**

About 69 detected insertions covered 16,440 bp of *M*. *destructor* genome and all the copies belong to the same lineage. Some of them have perfect TIRs with 84 bp of length flanking deleted ORFs whereas some others have complete ORF with the DDE domain but without TIRs.

- **MITEs**

The screening of MITEs by the REPET package enabled the identification of 83 lineages representing 5681 copies with a median size of 507 bp. TIR sizes are highly variable ranging from 17 to 777 bp. As TIR-flanked regions were noticeably short sizing 50 bp (Fig 3; S2 Table), it was hard to determine their corresponding superfamilies. Thus, MITE tracker was used, and 219563 putative MITEs were identified. The analysis of terminal TIRs and TSD sequences allowed the classification of 1237 MITE sequences into six Superfamilies. The *P* and TC1/mariner superfamilies were the most represented, with 515 and 312 MITEs, respectively, followed by the CACTA superfamily with 182 MITEs, then *Pif-Harbinger* and *hAT* with 80 and 79 elements, respectively. The *PiggyBac* superfamily was represented by only seven MITEs. The remaining sequences were unclassified.

*Helitrons*. *Helitrons* were identified via the conserved *helicase* domain enabling to annotate 220 copies, which belong to seven lineages and constitute 11% of the identified Class II elements (Fig 3 and S2 Table). Consensuses have sizes ranging from 600 to 2400 bp and similarities with *Helitrons* isolated from Drosophilidae and aquatic organisms like cnidarians *Hydra vulgaris* and lamprey *Petromyzon marinus*. They are characterized by a *Pif1*-like *helicase* domain belonging to the *P-loop_NTPase* superfamily at their C-terminal region.

*Mavericks*. *Mavericks* constitute 21% of the identified Class II elements annotated by 10 consensuses whose sizes vary from 600 to 4500 bp. The median size of 1243 bp reflect the fragmented state of the 408 Maverick copies with accumulated deletions. Indeed, *Mavericks* including the *Maverick/polintons* superfamily were considered as giant elements sized from 15 to 30 kb [73, 74]. The identified elements have only the DNA *Polymerase* or the *Integrase* domain.

#### noCat repeats

These unclassified repeats were distributed into 843 consensuses spanning about 11.93% of the genomic assembly and 70.48% of the covering repeatome. These consensuses were mostly short (70% are less than 1000bp). Moreover, the filtration step showed that only 30 noCats consensuses were generated by more than three copies. The blast of the 843 noCat consensuses against the identified TE library allowed the reclassification of 37 consensuses including one LTR, five LINEs, three SINEs, three retrotransposons, eight TIRs and 17 MITEs. The rest of the noCats could be parts of TEs which could not be fully generated because of the highly fragmented state of the draft genome of *M*. *destructor*. Indeed, 16,273 contigs were less than 1kb in length.

### Phylogenetic analyses

The identified TEs were aligned (S1 Appendix) then classified per superfamily. Among Class I retrotransposons, the *Ty3/gypsy* and *Bel-Pao* elements from the LTR order exhibited extensive diversifications (S1 and S2 Figs) compared to the *copia* and the Non-LTR elements (S3–S5 Figs). In Class II transposons, TIR elements, *Mavericks/Polintons* and *Helitrons* also showed a noticeable diversity among families and subfamilies (S6–S10 Figs). Among TIR transposons, *hAT* elements were grouped with plant *hAT* families in clusters supported by high bootstrap values (S8 Fig). Otherwise, *Mavericks* were clustered with TEs isolated from aquatic vertebrates and cnidarians (S10 Fig).

### TEs age estimation

**Fig 4:**
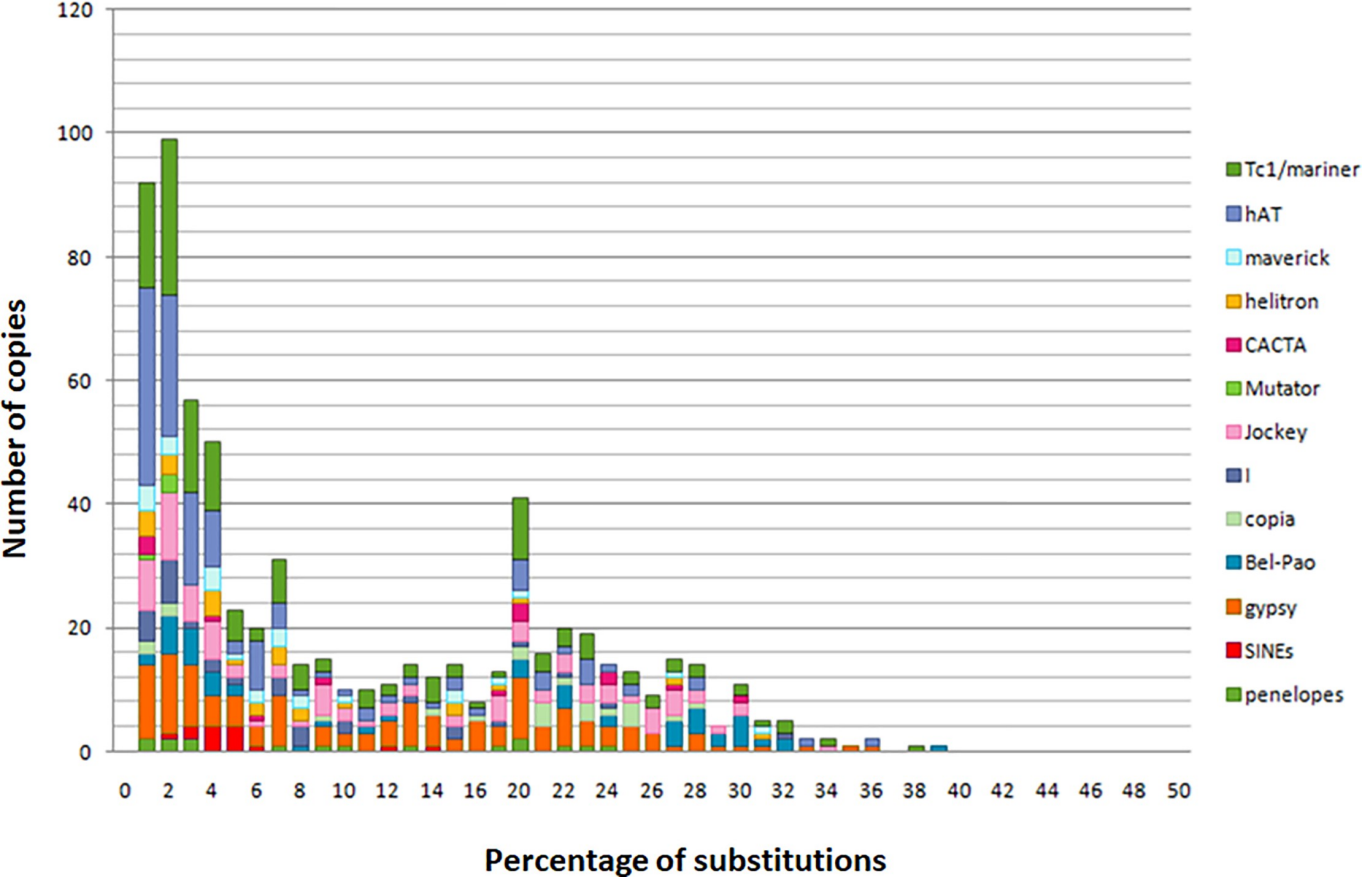
Estimate of TE ages and transposition events in *Mayetiola destructor* genome. The age of TEs is given as the percentage of substitutions from their consensus sequences shown in the x-axis. Recent events of transposition have low substitution levels. The y-axis shows the TEs’ copy number.

**Fig 5:**
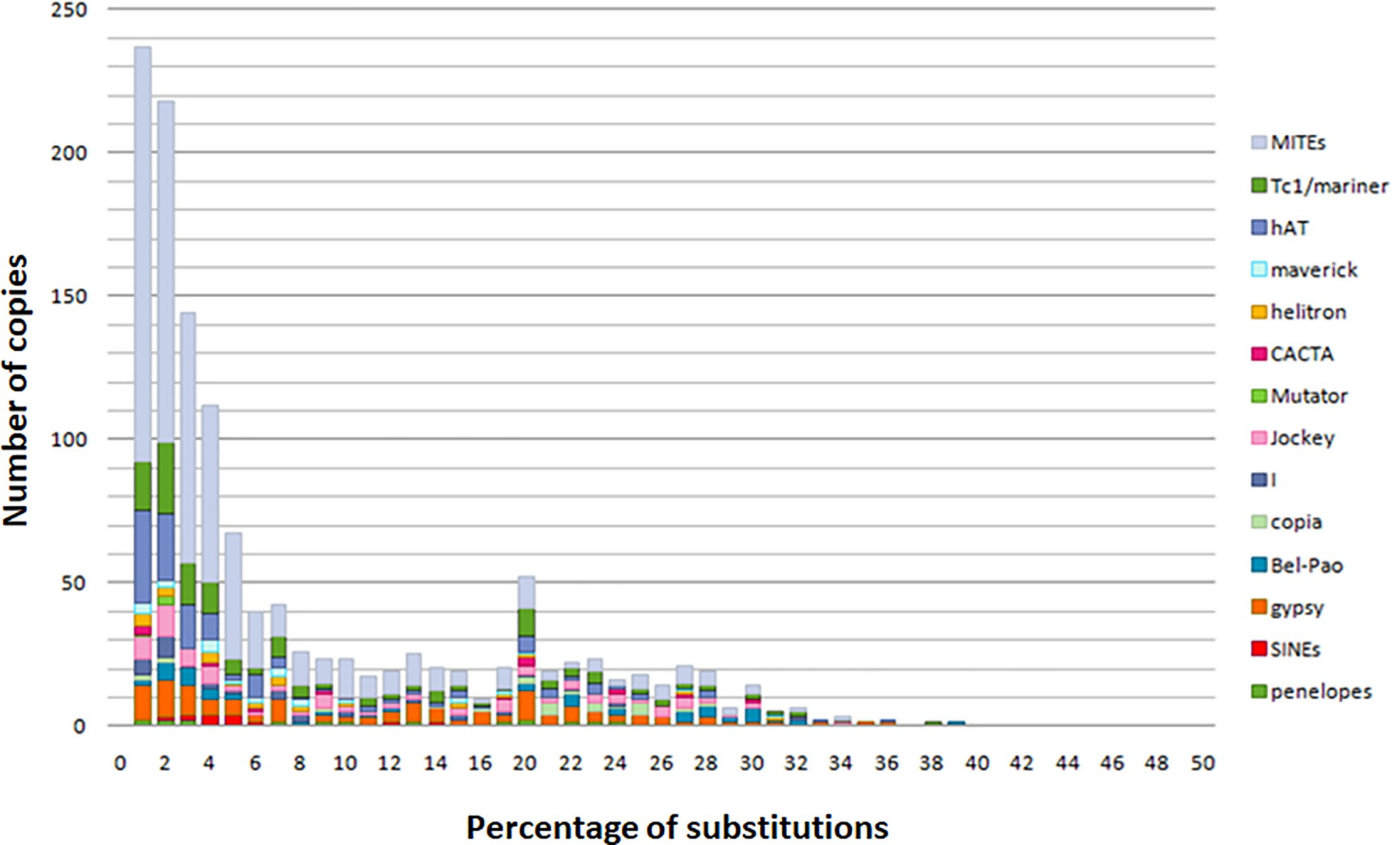
Estimate of MITEs and TEs transposition events in *Mayetiola destructor* genome. The age of TEs is given as the percentage of substitutions from their consensus sequences shown in the x-axis. Recent events of transposition have low substitution levels. The y-axis shows the TEs’ copy number. The age distributions suggest dominance MITEs activity between 1 and 6% of divergence.

TEs age estimation and dynamic studies were carried out based on the divergence rates using the terminal fork branch lengths from TE families’ phylogenies (Figs 4 and 5). The divergence estimation of copies from their consensuses (putative ancestral sequences) informs about the ages of these TEs and the divergence measures between copies of terminal fork make it possible to estimate the ages of the last transposition events. The more the divergence between close copies is important, the more the activity is ancient.

These estimations reveal the ages of last events give no information about a possible activity of the most recent copies. Age of TE activities was estimated in divergence units. Analysis revealed a first peak (from 1 to 2% of divergence from present) showing waves of TEs invasion recently occurring during the same period by several TE families through many bursts of transpositions. This period was characterized by the amplification of Class II elements which is likely more important than those of Class I.

About ¼ of these TEs belongs to the *hAT* superfamily followed by *Tc1/mariner* and *Gypsy* superfamilies. As for the *hAT*, many transposition events emerged significantly including at least nine bursts (between 1 and 2% of divergence from present) suggesting *M*. *destructor* specific TEs activities. As for *Tc1/mariner* and *Gypsy* elements, despite their recent explosion, their activity seems to be regular and common to other species since they are known to be widespread in eukaryotes including insects. This recent peak of invasions includes the emergence of new TE families such as *Mutator-like* elements with low copy numbers. This emergence suggests a very recent invasion of the genome of *M*. *destructor* by this Class II TE group or a resurrection of this superfamily to proliferate again after a long period of silencing.

Analysis revealed also a second peak at 20% of divergence from present showing ancient mobilization of many TE families, notably *Tc1/mariner* and *Gypsy* but less important than the most recent ones.

Activities of TEs belonging to *Bel-Pao*, *Jockey*, *Penelope*, *CACTA* and *Helitrons* superfamilies were found to be partially or completely extinct after a period of ancient transposition events at different times. However, they display more recent transposition events suggesting the reinvasion of the genome of *M*. *destructor* by these families through horizontal transfer, the reemergence of some lineages from defective copies or by their reactivation by *trans*-mobilization. *Copia* elements were extinct for a long period of time before to reemerge with a low copy number in the recent peak of bursts.

The number of bursts reflects the diversification of TE families; the more the number of consensuses is important the more TEs are diversified. *Tc1/mariner* and *hAT* elements seem to be more diversified than *Gypsy* elements.

The analysis of MITEs transposition events showed the same evolution as the other TEs with two peaks of bursts.

### TEs near genes

**Fig 6:**
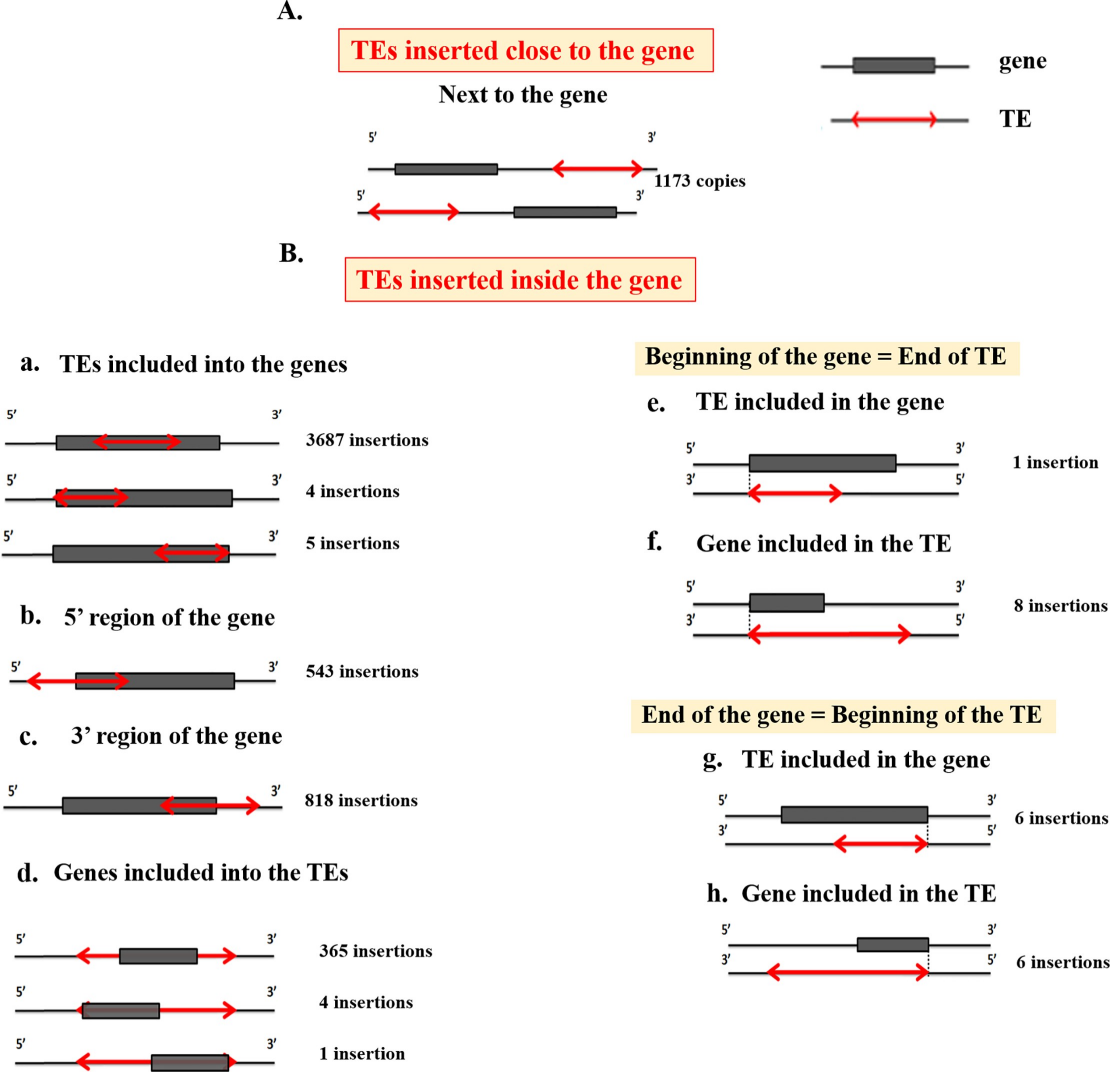
Schematic representation of the different TEs positions in the vicinity of the host genes of *M*. *destructor*. Genes and TEs are respectively indicated in grey rectangles and red double arrows. The number of insertions is indicated on the right of each diagram. The screening of TE insertions in genes involved in the Hessian fly virulence revealed insertions of a MITE and a *gypsy* element in SSGP genes which induce the formation of galls in the stem and the nutritive tissue of the host plants. The MITE was detected in the 5’ end at reverse orientation and exhibits a recent event of transposition at 0.61% of divergence whereas the *Gypsy* element dating at 0.07% was found in the reverse orientation of the SSGP gene in its 3’ region.

TE insertions in the vicinity of *M*. *destructor genes* were classified into two main categories: TEs inserted close or inside the gene (Fig 6; Table 2). The first category has 114 insertions at less than 2 kb from the gene. Different positions were however analysed in the second category with the most abundant into the first exon with 251 insertions. Few TE insertions overlap the 5’ and 3’ extremities with a significant difference in orientation on the 5’side (Chi-square test p-value = 0.05, ddl = 4). Some genes included inside the TEs, were excluded from analysis, and considered as TEs, which are not well annotated.

**Table 2:** Representation of the cases and numbers of TE among *Mayetiola destructor* genes.

Position			TE sense insertion	TE antisense insertion	Chi2-Test
**Outside of the gene**	Next to the gene		620	573	Non-significant
**Inside of the gene**	TEs included into the gene		1849	1847	Non-significant
	Genes included into the TEs		205	165	Significant
	5’ region of the gene		304	239	Significant
	3’ region of the gene		412	406	Non-significant
	Beginning of the gene = End of TE	TE included in the gene	0	9	-*
		Gene included in the TE			
	End of the gene = Beginning of the TE	TE included in the gene	0	12	-*
		Gene included in the TE			

*Chi-2 test was not performed for these positions as TEs orientations are already determined.

TEs having shown a recent activity at less than 2% of divergence were selected to study whether recent insertions occurred into or next to the host genes. We have revealed 86 such insertions from which 14 were inserted close to the genes. Among them 10 occurred at less than 2 kb and four at the 5’ side of host genes. Forty-one TEs were inserted inside genes, among which 21 TE copies had exonic insertions with 12 in the first CDS and 31 overlapping the 5’ or the 3’ side of the gene. Analyses of inserted TEs copies showed the prevalence of MITEs (66.67%), followed by *hAT* (17.86%), *Tc1/mariner* (9.52%) then the *Gypsy* and *mutator* elements with (4.76%) and (1.19%), respectively (Table 3).

**Table 3:** Categories of insertion sites and numbers of TEs having recently transposed (less than 2% of divergence).

Superfamily	Number of insertion sites	Next to genes	Included inside the gene		Gene 5’ side		Gene 3’ side	
***Ty3/gypsy***	4	0	0		3		1	
***hAT***	15	0	6 Exonic insertions		3		5	
			1 Intronic insertion					
***Tc1/mariner***	8	3 (5’ side)	2 Exonic insertions		0		0	
			3 Intronic insertions					
***Mutator***	1	0	0		1		0	
***MITEs***	58	10 (3’ side)	13 Exonic insertions		10		8	
		1 (5’ side)						
			16 Intronic insertions					
Total	86	14	Sens	Anti-sens	Sens	Anti-Sens	Sens	Anti-sens
			17	24	9	8	5	9

SSGP genes also show older TE insertions dating from 3% to 15% of divergence, and belonging to the different superfamilies *Gypsy*, *Bel-Pao*, *Jockey*, *Penelope*, *tc1/mariner*, *hAT*, *CACTA* and MITE*s*. These insertions were represented mainly by MITEs (34% of insertions) and *Gypsy* retrotransposons (28% of insertions).

Additionally, analyses of TE insertions in the chemosensory genes have shown nine insertions among which a single MITE copy having its last transposition activity at 12% of divergence is inserted in the same orientation as a gene encoding Gustatory Receptors (GRs). No recent activities have been shown for the other TEs that were inserted in genes encoding Olfactory Receptors (ORs).

## Discussion

### DNA transposons are more abundant than retrotransposable elements

The present study assesses the TE content in the sequenced genome of the Hessian fly. The *Mayetiola* genome is estimated to be 153Mb in length, and we reported that TEs make up 16.36% of its sequence. Similar proportions were estimated in other Dipteran genomes like the Drosophilidae species whose TE content varies between 3 and 25% [75].

Our evaluation of TE content is probably an underestimation and TEs may be obscured by the large “unclassified” repeated sequences found by our de novo approach. These repeated sequences, considered as noCats, could constitute new or highly divergent types of TEs that had been disregarded here [6]. Additionally, the WGS assembly may be missing some regions that are rich in repeated sequences like centromeres or other heterochromatic regions which could be parts of genomic gaps [76].

The comparison of genome proportions of TE classes showed that DNA transposons occur in higher amounts than Class I elements do. The situation is similar in the mosquito *Anopheles gambiae* [77], the hymenopteran Jerdon’s jumping ant *Harpegnathos* [6] and the coleopterans, *Tribolium castaneum* and *Anoplophora glabripennis* [78].

Analysis of TE composition revealed that TIR elements dominate the population of TEs in the *Mayetiola* genome followed by LTRs and Non-LTR. TIRs abundance has been also described quite recently in six tsetse fly species [79]. The prevalence of the TIR transposons may be related to the activity of such elements, which may depend on just a few active copies of the family. This hypothesis is supported by the presence of potentially active copies found in Tc1/mariner superfamily.

#### Waves of transposition explain the observed TE diversity

Transposition event date estimates in *M*. *destructor* revealed recent bursts which involved essentially DNA transposons. It has been reported by previous studies that Class II elements are active for short time periods then escape the defense systems of the host by horizontal transfer unlike retrotransposons which establish long-term associations with the host genome [80].

In *M*. *destructor*, the most recent bursts show an explosion of *hAT* elements with the emergence of new lineages, which would originate from horizontal transfer. Noticeably, in some Diptera species, different *hAT* families have shown similar activities such as *Herves* family in *Anopheles gambiae*, *Hermes* in *Musca domestica* and *Aedes aegypti* as well as *hobo* in Drosophilidae [81].

The identified MITE*s* in *M*. *destructor* are highly frequent which reflect their high level of transposition. The high abundance of MITEs was previously reported in several insect species with similarly high rates of activities [82]. The small size of MITEs would allow them to escape the defense system of the host genome leading therefore to their accumulation [89]. Most identified MITEs exhibited terminal inverted repeats, noticeably short ORFs that would come from relics of old DNA transposons after an Abortive Gap Repair [83–85].

Analysis of retrotransposons showed that, except for one *Copia* element, all the copies are inactive despite the polymorphism of their insertions and the large diversity of the identified LTR lineages. This could be explained by old activities of a high number of copies which invaded the species followed by inactivation processes and mutations [77, 86]. In addition, we cannot exclude the presence of an eventual active elements in *M*. *destructor* genome but these LTR sequences would be presumably missed because of eventual erroneous assembly of this insect WGS. Indeed, the genome sequences were assembled by combining Illumina and 454 sequence data which are characterized by short reads of 35 to 300 bp [87]. Therefore, these read sizes are impeding the complete reconstruction of genomes and can affect further analyses and mislead biological interpretations [88].

Among LTR retrotransposons, *Ty3/gypsy* elements showed a regular activity during a long period of time. It could be due to the cooperation of deleted copies whose products are complementary to catalyse the transposition process. According to Sabot and Schulman [89], a single copy containing at least one ORF or a complete domain can be considered as an autonomous element which would promote the amplification of defective elements leading to their expansion in *M*. *destructor* genome.

*Bel-Pao* and *copia* retrotransposons, have shown recent activities after their extinction. This reactivation would have taken place simultaneously with the invasion of new variants involving complete copies which may act in *trans* to mobilize the TEs of the other lineages [90].

The Non-LTR elements have shown a large fraction of copies with deleted 5’ regions. This has also been observed in *Anopheles funestus* and has been reported as a “Dead On Arrival” mechanism (DOA) when these elements lose a part of their 5’ region after an incomplete reverse transcription which in turn inhibit their transposition activity [81, 91] (Fernandez 2017, Han 2010). The activity profile of *Jockey* and *I* elements shows an old activity which has been reactivated recently (at 2% of divergence).

The study of the consensuses representing TE lineages has shown that Class II elements contain more consensuses than Class I elements, thus reflecting the diversity of DNA transposon lineages in *M*. *destructor* genome. These results are in line with prior studies on insects comparing TEs repertoires which showed that DNA transposons are more diverse than retrotransposons [92].

Phylogenetic analyses of the different superfamilies identified in the *M*. *destructor* genome have shown different types of consensuses representing diverse lineages and families. Some of these groups have already been identified in other species and some others formed new groups suggesting new classifications. A part of the observed TEs diversity could be due to the small size of the Hessian fly genome as shown by Elliot et al [93].

Phylogenetic trees revealed that several identified TEs were found to be originated from species from other kingdoms which could be explained by the lateral movements of transposons across eukaryotic phyla. This Horizontal transfer (HT) was previously reported in many taxa including insects with plants, crustaceans, and cnidarians [94–96].

#### Possible role of TEs in *M*. *destructor* adaptation

The identified TE insertion sites were investigated for their possible involvement in certain key genes regulations as those related to the pest virulence. Elements inserted within 2 kb of the gene have been particularly studied because at this distance, TE insertion may influence the *cis* regulation of the gene expression [97].

On the one hand, most insertions were revealed inside introns or covering a part of the exon and the nearby intron. Insertions in the introns could have no impact since these regions are characterized by high levels of mutations and TEs should accumulate different mutations by neutral selection [11]. Nevertheless, insertions covering the exon-intron junction could involve modifications of the splice sites within the insect [98, 99]. Further in-depth functional studies would be needed to confirm these impacts on the studied genes.

On the other hand, a low number of insertions occurred in the exons as well as the 5’and 3’ extremities. This could be explained by a purifying selection leading to the removal of these elements to protect the coding region and the regulatory domains corresponding to gene promoters and terminators [97, 98]. However, a significant difference between insertion orientations has been shown in the 5’ regions of host genes in favor of the sense insertion which could lead to a disturbance of the gene expression [100, 101] whereas the antisense insertions could generate transcripts interfering with sense transcripts [98].

TE insertions in the vicinity of virulence and chemosensory genes occurred more frequently in the SSGP virulence effector genes. This could be explained by a higher selective pressure on the chemosensory genes for their important role in the life cycle of this pest, mainly in the adult stage which is noticeably short and requires the functionality of these genes for finding the partner, mating, and laying eggs [37].

Recent events of transposition of *Ty3/gypsy* elements and MITEs in genes encoding SSGP proteins is consistent with researches led by Wessler et al. [102] and Casacuberta & Santiago [103] who noted an association between LTR elements and MITEs and the host genes contributing to their evolution.

Hence, the mobilome dynamic in the Hessian fly genome originates from the interaction between the multitude of coexisting TE lineages and families reflecting different evolutionary scenarios. Consequently, the inactive forms of certain lineages of Class I retrotransposons may cooperate to ensure their amplification despite the mutations they have accumulated, while the non-autonomous Class II transposons would ensure their activity by *trans*-mobilization using potentially active copies.

## Conclusions

In this work, we performed a Genome-Wide bioinformatics Scanning of transposable elements in Hessian fly. TEs have shown a large diversity with different waves of invasions and activities. Some elements were inserted in the vicinity of host genes that may be important for adaptation. Therefore, the analyses carried out constitute a crucial step for subsequent in-depth studies focusing on promoter, end of transcription signals, as well as splicing signals originated from TEs, to better estimate their impact on the virulence genes of this insect pest.

## Supporting information

S1 Table Coverage of TE orders per chromosome in *M*. *destructor*. **Un**: unplaced scaffolds; **DHX**: *Helitrons*; **DMX**: *Maverick*; **DTX**: TIRs elements; **DXX**: Other Class II transposons; **RIX**: LINEs; **RLX**: LTR retrotransposons; **RSX**: SINEs; **RXX_TRIM**: Terminal Repeat Transposons in Miniature; **RPX**: *Penelopes*.
(DOCX)

S2 Table Distribution of TE superfamilies and the other repetitive sequences identified in the genome of *Mayetiola destructor*. (DOCX)

S1 Appendix Fasta file of nucleotide sequences alignment per TE superfamily in *Mayetiola destrucor* with TE references. (TXT)

S1 Fig Classification of the 22 consensuses *Ty3 / Gypsy* superfamily of *Mayetiola destructor*. The consensuses identified in *Mayetiola destructor* are marked by triangles. Bootstrap values less than 50% are eliminated. The tree is built by the ML method (model HKY85) with a bootstrap of 1000 repetitions.
(DOCX)

S2 Fig Classification of the 12 consensuses of the *Bel-Pao* superfamily of *Mayetiola destructor*. The consensuses identified in *Mayetiola destructor* are marked by triangles. Bootstrap values less than 50% are eliminated. The tree is built by the ML method (model HKY85) with a bootstrap of 1000 repetitions. The sequences marked with colored triangles represent the different lines identified in *Mayetiola destructor*.
(DOCX)

S3 Fig Classification of the three-consensus superfamily *Ty1 / Copia* of *Mayetiola destructor*. The consensuses identified in *Mayetiola destructor* are marked by triangles. Bootstrap values less than 50% are eliminated. The tree is built by the ML method (model HKY85) with a bootstrap of 1000 repetitions. The sequences marked with colored triangles represent the different lines identified in *Mayetiola destructor*.
(DOCX)

S4 Fig Classification of the 14 consensuses of the *Jockey* superfamily of *Mayetiola destructor*. The consensuses identified in *Mayetiola destructor* are marked by triangles. Bootstrap values less than 50% are eliminated. The tree is built by the ML method (model HKY85) with a bootstrap of 1000 repetitions. The sequences marked with colored triangles represent the different lines identified in *Mayetiola destructor*.
(DOCX)

S5 Fig Classification of the four-consensus superfamily *I* of *Mayetiola destructor*. The consensuses identified in *Mayetiola destructor* are marked by triangles. Bootstrap values less than 50% are eliminated. The tree is built by the ML method (model HKY85) with a bootstrap of 1000 repetitions. The sequences marked with colored triangles represent the different lines identified in *Mayetiola destructor*.
(DOCX)

S6 Fig Classification of the 28 consensuses *Tc1 / mariner* superfamily of *Mayetiola destructor*. The consensuses identified in *Mayetiola destructor* are marked by triangles. Bootstrap values less than 50% are eliminated. The tree is built by the ML method (model HKY85) with a bootstrap of 1000 repetitions. The clades of *mariner*-like elements (MLEs), *Tc1*-like elements (TLEs) and *pogo*-like elements are highlighted in pink, green and orange, respectively. The three shades in the MLEs clade refer to the *mauritiana*, *irritans* and *rosa* subfamilies from the top to the bottom.
(DOCX)

S7 Fig Classification of the 24 consensuses of *Mayetiola destructor hAT* superfamily within isolated groups in insects. The consensuses identified in *Mayetiola destructor* are marked by triangles. Bootstrap values less than 50% are eliminated. The tree is built by the ML method (model HKY85) with a bootstrap of 1000 repetitions. The colored triangles represent the *hAT* lines identified in *Mayetiola destructor*.
(DOCX)

S8 Fig Classification of *Mayetiola destructor hAT* superfamily elements within isolated groups in plants and fungi. The consensuses identified in *Mayetiola destructor* are marked by triangles. Bootstrap values less than 50% are eliminated. The tree is built by the ML method (model HKY85) with a bootstrap of 1000 repetitions.
(DOCX)

S9 Fig Phylogeny of the seven consensus *Helitrons* of *Mayetiola destructor*. The consensuses identified in *Mayetiola destructor* are marked by triangles. Bootstrap values less than 50% are eliminated. The tree is built by the ML method (model HKY85) with a bootstrap of 1000 repetitions. The reference sequences are isolated from the following species: AG: *Anopheles gambiae*; DF: *Drosophila ficusphila*; DBP: *Drosophila bipectinata*; LMi: *Locusta migratoria*; HRD: *Drosophila rhopaloa*; CMi: *Callorhinchusmilii*; SPur: *Strongylocentrotus purpuratus*; SP: *Strongy locentrotus purpuratus*; OS: *Oryza sativa*.
(DOCX)

S10 Fig Phylogeny of *Maverick / Polintons* elements of *Mayetiola destructor*. The consensuses identified in *Mayetiola destructor* are marked by triangles. Bootstrap values less than 50% are eliminated. The tree is built by the ML method (model HKY85) with a bootstrap of 1000 repetitions. The reference sequences are isolated from the following species: DR: *Danio rerio*; DBP: *Drosophila bipectinata*; NV: *Nematostella vectensis*; NVi: *Nasonia vitripennis*; TC: *Tribolium castaneum*; DEu: *Drosophila eugracilis*; DK: *Drosophila kikkawai*.
(DOCX)

## References

[1] Bourque G., et al., Ten things you should know about transposable elements. Genome Biology, 2018. 19(1): p. 199. doi: 10.1186/s13059-018-1577-z 30454069 PMC6240941

[2] Kazazian H.H. Jr., Mobile elements: drivers of genome evolution. Science, 2004. 303(5664): p. 1626–32. doi: 10.1126/science.1089670 15016989

[3] Platt R.N. 2nd, Vandewege M.W., and Ray D.A., Mammalian transposable elements and their impacts on genome evolution. Chromosome research: an international journal on the molecular, supramolecular and evolutionary aspects of chromosome biology, 2018. 26(1–2): p. 25–43. doi: 10.1007/s10577-017-9570-z 29392473 PMC5857283

[4] Finnegan D.J., Eukaryotic transposable elements and genome evolution. Trends Genet, 1989. 5(4): p. 103–7. doi: 10.1016/0168-9525(89)90039-5 2543105

[5] Guio L. and Gonzalez J., New Insights on the Evolution of Genome Content: Population Dynamics of Transposable Elements in Flies and Humans. Methods Mol Biol, 2019. 1910: p. 505–530. doi: 10.1007/978-1-4939-9074-0_16 31278675

[6] Petersen M., et al., Diversity and evolution of the transposable element repertoire in arthropods with particular reference to insects. BMC Evol Biol, 2019. 19(1): p. 11. doi: 10.1186/s12862-018-1324-9 30626321 PMC6327564

[7] Kelley J.L., et al., Compact genome of the Antarctic midge is likely an adaptation to an extreme environment. Nature communications, 2014. 5(1): p. 1–8. 10.1038/ncomms5611 PMC4164542 25118180

[8] Burns K.H. and Boeke J.D., Human transposon tectonics. Cell, 2012. 149(4): p. 740–52. doi: 10.1016/j.cell.2012.04.019 22579280 PMC3370394

[9] Casals F., Cáceres M., and Ruiz A., The Foldback-like Transposon Galileo Is Involved in the Generation of Two Different Natural Chromosomal Inversions of Drosophila buzzatii. Molecular Biology and Evolution, 2003. 20(5): p. 674–685. doi: 10.1093/molbev/msg070 12679549

[10] Chuong E.B., Elde N.C., and Feschotte C., Regulatory activities of transposable elements: from conflicts to benefits. Nat Rev Genet, 2017. 18(2): p. 71–86. doi: 10.1038/nrg.2016.139 27867194 PMC5498291

[11] Hirsch C.D. and Springer N.M., Transposable element influences on gene expression in plants. Biochim Biophys Acta Gene Regul Mech, 2017. 1860(1): p. 157–165. doi: 10.1016/j.bbagrm.2016.05.010 27235540

[12] Rebollo R., Farivar S., and Mager D.L., C-GATE—catalogue of genes affected by transposable elements. Mobile DNA, 2012. 3(1): p. 9–9. doi: 10.1186/1759-8753-3-9 22621612 PMC3472293

[13] González J., et al., High rate of recent transposable element-induced adaptation in Drosophila melanogaster. PLoS biology, 2008. 6(10): p. e251–e251. doi: 10.1371/journal.pbio.0060251 18942889 PMC2570423

[14] Jin G.H., Zhou Y.L., Yang H., Hu Y.T., Shi Y., Li L., et al. Genetic innovations: Transposable element recruitment and de novo formation lead to the birth of orphan genes in the rice genome. Journal of Systematics and Evolution., 2019.

[15] Joly-Lopez Z. and Bureau T.E., Exaptation of transposable element coding sequences. Curr Opin Genet Dev, 2018. 49: p. 34–42. doi: 10.1016/j.gde.2018.02.011 29525543

[16] Itokawa K., et al., Genomic structures of Cyp9m10 in pyrethroid resistant and susceptible strains of Culex quinquefasciatus. Insect Biochem Mol Biol, 2010. 40(9): p. 631–40. doi: 10.1016/j.ibmb.2010.06.001 20600899

[17] Ding Y., et al., Natural courtship song variation caused by an intronic retroelement in an ion channel gene. Nature, 2016. 536(7616): p. 329–32. doi: 10.1038/nature19093 27509856

[18] González J., et al., Genome-wide patterns of adaptation to temperate environments associated with transposable elements in Drosophila. PLoS Genet, 2010. 6(4): p. e1000905. doi: 10.1371/journal.pgen.1000905 20386746 PMC2851572

[19] Berthelier J., et al., Pirate: a pipeline to retrieve and annotate transposable elements in tisochrysis lutea genome. Phycologia, 2017. 56(4): p. 18.

[20] Flutre T., et al., Considering transposable element diversification in de novo annotation approaches. PLoS One, 2011. 6(1): p. e16526. doi: 10.1371/journal.pone.0016526 21304975 PMC3031573

[21] Flynn J.M., et al., RepeatModeler2 for automated genomic discovery of transposable element families. Proc Natl Acad Sci U S A, 2020. 117(17): p. 9451–9457. doi: 10.1073/pnas.1921046117 32300014 PMC7196820

[22] Tempel S., Using and understanding RepeatMasker, in Mobile Genetic Elements. 2012, Springer. p. 29–51. 10.1007/978-1-61779-603-6_2 22367864

[23] Kennedy R.C., et al., An automated homology-based approach for identifying transposable elements. BMC Bioinformatics, 2011. 12: p. 130. doi: 10.1186/1471-2105-12-130 21535899 PMC3107183

[24] Kofler R., Gómez-Sánchez D., and Schlötterer C., PoPoolationTE2: comparative population genomics of transposable elements using Pool-Seq. Molecular biology and evolution, 2016. 33(10): p. 2759–2764. doi: 10.1093/molbev/msw137 27486221 PMC5026257

[25] Hoen D.R., et al., A call for benchmarking transposable element annotation methods. Mob DNA, 2015. 6: p. 13. doi: 10.1186/s13100-015-0044-6 26244060 PMC4524446

[26] Lerat E., Identifying repeats and transposable elements in sequenced genomes: how to find your way through the dense forest of programs. Heredity (Edinb), 2010. 104(6): p. 520–33. doi: 10.1038/hdy.2009.165 19935826

[27] Ou S., et al., Benchmarking transposable element annotation methods for creation of a streamlined, comprehensive pipeline. Genome Biology, 2019. 20(1): p. 275. doi: 10.1186/s13059-019-1905-y 31843001 PMC6913007

[28] Klai K., et al., Screening of Helicoverpa armigera Mobilome Revealed Transposable Element Insertions in Insecticide Resistance Genes. Insects, 2020. 11(12). doi: 10.3390/insects11120879 33322432 PMC7764229

[29] Ananiev E.V. and Ilyin Y.V., A comparative study of the location of mobile dispersed genes in salivary gland and midgut polytene chromosomes of Drosophila melanogaster. Chromosoma, 1981. 82(3): p. 429–35. doi: 10.1007/BF00285767 6262030

[30] Flanders K.L., et al., Biology and management of Hessian fly in the Southeast. 2013.

[31] Schmid R.B., et al., Hessian fly (Diptera: Cecidomyiidae) biology and management in wheat. Journal of Integrated Pest Management, 2018. 9(1): p. 14.

[32] Smiley R.W., et al., Economic impact of Hessian fly (Diptera: Cecidomyiidae) on spring wheat in Oregon and additive yield losses with Fusarium crown rot and lesion nematode. J Econ Entomol, 2004. 97(2): p. 397–408. doi: 10.1093/jee/97.2.397 15154461

[33] Stuart J.J., et al., Gall midges (Hessian flies) as plant pathogens. Annual review of phytopathology, 2012. 50: p. 339–357. doi: 10.1146/annurev-phyto-072910-095255 22656645

[34] Buntin D.G. and Chapin J.W., Biology of Hessian fly (Diptera: Cecidomyiidae) in the southeastern United States: geographic variation and temperature-dependent phenology. Journal of economic entomology, 1990. 83(3): p. 1015–1024.

[35] Cherif A., et al., Distribution, population dynamics and damage of Hessian fly, Mayetiola destructor (Diptera: Cecidomyiidae) in North Tunisia. Journal of Applied Entomology, 2020.

[36] Lhaloui S., et al., Les cécidomyies des céréales au Maroc: Biologie, dégâts et moyens de lutte. INRA, Maroc, 2006: p. 1–5.

[37] Harris M.O., et al., Grasses and gall midges: plant defense and insect adaptation. Annu Rev Entomol, 2003. 48: p. 549–77. doi: 10.1146/annurev.ento.48.091801.112559 12460937

[38] Zhao C., et al., Avirulence gene mapping in the Hessian fly (Mayetiola destructor) reveals a protein phosphatase 2C effector gene family. J Insect Physiol, 2016. 84: p. 22–31. doi: 10.1016/j.jinsphys.2015.10.001 26439791

[39] Hatchett J. and Gallun R.L., Genetics of the ability of the Hessian fly, Mayetiola destructor, to survive on wheats having different genes for resistance. Annals of the Entomological Society of America, 1970. 63(5): p. 1400–1407.

[40] Chen M.-S., et al., A super-family of genes coding for secreted salivary gland proteins from the Hessian fly, Mayetiola destructor. Journal of insect science (Online), 2006. 6: p. 1–13. doi: 10.1673/2006.06.12.1 19537963 PMC2990301

[41] BEN AMARA W., et al., An overview of irritans-mariner transposons in two Mayetiola species (Diptera: Cecidomyiidae). EJE, 2017. 114(1): p. 379–390.

[42] Russell V.W. and Shukle R.H., Molecular and cytological analysis of a mariner transposon from Hessian fly. J Hered, 1997. 88(1): p. 72–6. doi: 10.1093/oxfordjournals.jhered.a023062 9048446

[43] Shukle R.H. and Russell V.W., Mariner transposase-like sequences from the Hessian fly, Mayetiola destructor. J Hered, 1995. 86(5): p. 364–8. doi: 10.1093/oxfordjournals.jhered.a111604 7560872

[44] Behura S.K., Shukle R.H., and Stuart J.J., Assessment of structural variation and molecular mapping of insertion sites of Desmar-like elements in the Hessian fly genome. Insect Molecular Biology, 2010. 19(6): p. 707–715. doi: 10.1111/j.1365-2583.2010.01028.x 20636348

[45] Zhao C., et al., A massive expansion of effector genes underlies gall-formation in the wheat pest Mayetiola destructor. Curr Biol, 2015. 25(5): p. 613–20. doi: 10.1016/j.cub.2014.12.057 25660540

[46] Quesneville H., Nouaud D., and Anxolabehere D., Detection of new transposable element families in Drosophila melanogaster and Anopheles gambiae genomes. J Mol Evol, 2003. 57 Suppl 1: p. S50–9. doi: 10.1007/s00239-003-0007-2 15008403

[47] Bao Z. and Eddy S.R., Automated de novo identification of repeat sequence families in sequenced genomes. Genome Res, 2002. 12(8): p. 1269–76. doi: 10.1101/gr.88502 12176934 PMC186642

[48] Edgar R.C. and Myers E.W., PILER: identification and classification of genomic repeats. Bioinformatics, 2005. 21 Suppl 1: p. i152–8. doi: 10.1093/bioinformatics/bti1003 15961452

[49] Jurka J., et al., Repbase Update, a database of eukaryotic repetitive elements. Cytogenet Genome Res, 2005. 110(1–4): p. 462–7. doi: 10.1159/000084979 16093699

[50] Punta M., et al., The Pfam protein families database. Nucleic Acids Res, 2012. 40(Database issue): p. D290–301. doi: 10.1093/nar/gkr1065 22127870 PMC3245129

[51] Hoede C., et al., PASTEC: an automatic transposable element classification tool. PLoS One, 2014. 9(5): p. e91929. doi: 10.1371/journal.pone.0091929 24786468 PMC4008368

[52] Wicker T., et al., A unified classification system for eukaryotic transposable elements. Nat Rev Genet, 2007. 8(12): p. 973–82. doi: 10.1038/nrg2165 17984973

[53] Smit A.F.A., Hubley R., and Green P. 2013–2015; Repeat Masker 3.2.6]. Available from: http://www.repeatmasker.org.

[54] Jamilloux V., et al., De novo annotation of transposable elements: tackling the fat genome issue. Proceedings of the IEEE, 2016. 105(3): p. 474–481.

[55] Crescente J.M., et al., MITE Tracker: an accurate approach to identify miniature inverted-repeat transposable elements in large genomes. BMC Bioinformatics, 2018. 19(1): p. 348. doi: 10.1186/s12859-018-2376-y 30285604 PMC6171319

[56] Rognes T., et al., VSEARCH: a versatile open source tool for metagenomics. PeerJ, 2016. 4: p. e2584. doi: 10.7717/peerj.2584 27781170 PMC5075697

[57] Clark K., et al., GenBank. Nucleic Acids Res, 2016. 44(D1): p. D67–72. doi: 10.1093/nar/gkv1276 26590407 PMC4702903

[58] Bao W., Kojima K.K., and Kohany O., Repbase Update, a database of repetitive elements in eukaryotic genomes. Mob DNA, 2015. 6: p. 11. doi: 10.1186/s13100-015-0041-9 26045719 PMC4455052

[59] Hasegawa M., Kishino H., and Yano T., Dating of the human-ape splitting by a molecular clock of mitochondrial DNA. J Mol Evol, 1985. 22(2): p. 160–74. doi: 10.1007/BF02101694 3934395

[60] Kumar S., et al., MEGA X: Molecular Evolutionary Genetics Analysis across Computing Platforms. Mol Biol Evol, 2018. 35(6): p. 1547–1549. doi: 10.1093/molbev/msy096 29722887 PMC5967553

[61] Letunic I. and Bork P., Interactive Tree Of Life (iTOL): an online tool for phylogenetic tree display and annotation. Bioinformatics, 2007. 23(1): p. 127–8. doi: 10.1093/bioinformatics/btl529 17050570

[62] Fiston-Lavier A.-S., Vejnar C.E., and Quesneville H., Transposable element sequence evolution is influenced by gene context. arXiv preprint arXiv:1209.0176, 2012.

[63] Quesneville H., Twenty years of transposable element analysis in the Arabidopsis thaliana genome. Mob DNA, 2020. 11: p. 28. doi: 10.1186/s13100-020-00223-x 32742313 PMC7385966

[64] Needleman S.B. and Wunsch C.D., A general method applicable to the search for similarities in the amino acid sequence of two proteins. J Mol Biol, 1970. 48(3): p. 443–53. doi: 10.1016/0022-2836(70)90057-4 5420325

[65] Guindon S., et al., New algorithms and methods to estimate maximum-likelihood phylogenies: assessing the performance of PhyML 3.0. Syst Biol, 2010. 59(3): p. 307–21. doi: 10.1093/sysbio/syq010 20525638

[66] Guindon S. and Gascuel O., A simple, fast, and accurate algorithm to estimate large phylogenies by maximum likelihood. Syst Biol, 2003. 52(5): p. 696–704. doi: 10.1080/10635150390235520 14530136

[67] Quinlan A.R., BEDTools: the Swiss‐army tool for genome feature analysis. Current protocols in bioinformatics, 2014. 47(1): p. 11.12. 1–11.12. 34. doi: 10.1002/0471250953.bi1112s47 25199790 PMC4213956

[68] Quinlan A.R. and Hall I.M., BEDTools: a flexible suite of utilities for comparing genomic features. Bioinformatics, 2010. 26(6): p. 841–842. doi: 10.1093/bioinformatics/btq033 20110278 PMC2832824

[69] Poelchau M., et al., The i5k Workspace@NAL—enabling genomic data access, visualization and curation of arthropod genomes. Nucleic Acids Res, 2015. 43(Database issue): p. D714–9. doi: 10.1093/nar/gku983 25332403 PMC4384035

[70] Lee E., et al., Web Apollo: a web-based genomic annotation editing platform. Genome Biol, 2013. 14(8): p. R93. doi: 10.1186/gb-2013-14-8-r93 24000942 PMC4053811

[71] Robinson J.T., et al., Integrative genomics viewer. Nat Biotechnol, 2011. 29(1): p. 24–6. doi: 10.1038/nbt.1754 21221095 PMC3346182

[72] Thorvaldsdottir H., Robinson J.T., and Mesirov J.P., Integrative Genomics Viewer (IGV): high-performance genomics data visualization and exploration. Brief Bioinform, 2013. 14(2): p. 178–92. doi: 10.1093/bib/bbs017 22517427 PMC3603213

[73] Pritham E.J., Putliwala T., and Feschotte C., Mavericks, a novel class of giant transposable elements widespread in eukaryotes and related to DNA viruses. Gene, 2007. 390(1–2): p. 3–17. doi: 10.1016/j.gene.2006.08.008 17034960

[74] Haapa-Paananen S., Wahlberg N., and Savilahti H., Phylogenetic analysis of Maverick/Polinton giant transposons across organisms. Mol Phylogenet Evol, 2014. 78: p. 271–4. doi: 10.1016/j.ympev.2014.05.024 24882428

[75] Drosophila 12 Genomes, C., et al., Evolution of genes and genomes on the Drosophila phylogeny. Nature, 2007. 450(7167): p. 203–18. doi: 10.1038/nature06341 17994087

[76] Wang Z., et al., The genome of flax (Linum usitatissimum) assembled de novo from short shotgun sequence reads. Plant J, 2012. 72(3): p. 461–73. doi: 10.1111/j.1365-313X.2012.05093.x 22757964

[77] Fernandez-Medina R.D., Struchiner C.J., and Ribeiro J.M., Novel transposable elements from Anopheles gambiae. BMC Genomics, 2011. 12: p. 260. doi: 10.1186/1471-2164-12-260 21605407 PMC3212995

[78] Gilbert C., Peccoud J., and Cordaux R., Transposable Elements and the Evolution of Insects. Annu Rev Entomol, 2020. doi: 10.1146/annurev-ento-070720-074650 32931312

[79] Attardo G.M., et al., Comparative genomic analysis of six Glossina genomes, vectors of African trypanosomes. Genome Biol, 2019. 20(1): p. 187. doi: 10.1186/s13059-019-1768-2 31477173 PMC6721284

[80] Eickbush T.H., Retrotranspososns. Encyclopedia of Genetics., ed. Press A. 2001.

[81] Fernandez-Medina R.D., et al., Transposable elements in the Anopheles funestus transcriptome. Genetica, 2017. 145(3): p. 275–293. doi: 10.1007/s10709-017-9964-z 28424974 PMC5584644

[82] Yang G., et al., Tuned for transposition: molecular determinants underlying the hyperactivity of a Stowaway MITE. Science, 2009. 325(5946): p. 1391–4. doi: 10.1126/science.1175688 19745152

[83] Fattash I., et al., Miniature inverted-repeat transposable elements: discovery, distribution, and activity. Genome, 2013. 56(9): p. 475–86. doi: 10.1139/gen-2012-0174 24168668

[84] Feschotte C., Zhang X., and Wessler S.R., Miniature inverted-repeat transposable elements and their relationship to established DNA transposons, in Mobile DNA II. 2002, American Society of Microbiology. p. 1147–1158.

[85] Rubin E. and Levy A.A., Abortive gap repair: underlying mechanism for Ds element formation. Mol Cell Biol, 1997. 17(11): p. 6294–302. doi: 10.1128/MCB.17.11.6294 9343390 PMC232480

[86] Tubio J.M., Naveira H., and Costas J., Structural and evolutionary analyses of the Ty3/gypsy group of LTR retrotransposons in the genome of Anopheles gambiae. Mol Biol Evol, 2005. 22(1): p. 29–39. doi: 10.1093/molbev/msh251 15356275

[87] van Dijk E.L., et al., Ten years of next-generation sequencing technology. Trends Genet, 2014. 30(9): p. 418–26. doi: 10.1016/j.tig.2014.07.001 25108476

[88] Peona V., et al., Identifying the causes and consequences of assembly gaps using a multiplatform genome assembly of a bird-of-paradise. Mol Ecol Resour, 2020. 21(1): p. 263–286. doi: 10.1111/1755-0998.13252 32937018 PMC7757076

[89] Sabot F. and Schulman A.H., Parasitism and the retrotransposon life cycle in plants: a hitchhiker’s guide to the genome. Heredity (Edinb), 2006. 97(6): p. 381–8. doi: 10.1038/sj.hdy.6800903 16985508

[90] Blumenstiel J.P., Birth, School, Work, Death, and Resurrection: The Life Stages and Dynamics of Transposable Element Proliferation. Genes (Basel), 2019. 10(5). 10.3390/genes10050336 PMC6562965 31058854

[91] Han J.S., Non-long terminal repeat (non-LTR) retrotransposons: mechanisms, recent developments, and unanswered questions. Mob DNA, 2010. 1(1): p. 15. doi: 10.1186/1759-8753-1-15 20462415 PMC2881922

[92] Hua-Van A., et al., The struggle for life of the genome’s selfish architects. Biology direct, 2011. 6(1): p. 1–29. doi: 10.1186/1745-6150-6-19 21414203 PMC3072357

[93] Elliott T.A. and Gregory T.R., Do larger genomes contain more diverse transposable elements? BMC evolutionary biology, 2015. 15(1): p. 1–10. doi: 10.1186/s12862-015-0339-8 25896861 PMC4438587

[94] Grace C.A. and Carr M., The evolutionary history of mariner elements in stalk-eyed flies reveals the horizontal transfer of transposons from insects into the genome of the cnidarian Hydra vulgaris. PLoS One, 2020. 15(7): p. e0235984. doi: 10.1371/journal.pone.0235984 32658920 PMC7357744

[95] Dupeyron M., et al., Horizontal transfer of transposons between and within crustaceans and insects. Mobile DNA, 2014. 5(1): p. 4. doi: 10.1186/1759-8753-5-4 24472097 PMC3922705

[96] Lin X., Faridi N., and Casola C., An Ancient Transkingdom Horizontal Transfer of Penelope-Like Retroelements from Arthropods to Conifers. Genome Biol Evol, 2016. 8(4): p. 1252–66. doi: 10.1093/gbe/evw076 27190138 PMC4860704

[97] Cridland J.M., Thornton K.R., and Long A.D., Gene expression variation in Drosophila melanogaster due to rare transposable element insertion alleles of large effect. Genetics, 2015. 199(1): p. 85–93. 25335504 10.1534/genetics.114.170837 PMC4286695

[98] Casacuberta E. and Gonzalez J., The impact of transposable elements in environmental adaptation. Mol Ecol, 2013. 22(6): p. 1503–17. doi: 10.1111/mec.12170 23293987

[99] Zhang Y., Romanish M.T., and Mager D.L., Distributions of transposable elements reveal hazardous zones in mammalian introns. PLoS Comput Biol, 2011. 7(5): p. e1002046. doi: 10.1371/journal.pcbi.1002046 21573203 PMC3088655

[100] van de Lagemaat L.N., Medstrand P., and Mager D.L., Multiple effects govern endogenous retrovirus survival patterns in human gene introns. Genome Biol, 2006. 7(9): p. R86. doi: 10.1186/gb-2006-7-9-r86 17005047 PMC1794541

[101] Zhou M., Zhou Q., and Hänninen H., The distribution of transposable elements (TEs) in the promoter regions of moso bamboo genes and its influence on downstream genes. Trees, 2018. 32(2): p. 525–537.

[102] Wessler S.R., Bureau T.E., and White S.E., LTR-retrotransposons and MITEs: important players in the evolution of plant genomes. Curr Opin Genet Dev, 1995. 5(6): p. 814–21. doi: 10.1016/0959-437x(95)80016-x 8745082

[103] Casacuberta J.M. and Santiago N., Plant LTR-retrotransposons and MITEs: control of transposition and impact on the evolution of plant genes and genomes. Gene, 2003. 311: p. 1–11. doi: 10.1016/s0378-1119(03)00557-2 12853133

