# Supplementary material for: A Genomic Survey of Mayetiola destructor Mobilome Provides New Insights into the Evolutionary History of Transposable Elements in the Cecidomyiid Midges
Source: PLoS One. 2021 Oct 11;16(10):e0257996. doi: 10.1371/journal.pone.0257996 (PMC8504770; doi:10.1371/journal.pone.0257996)
Supplement: S1 Table — Un: unplaced scaffolds; DHX: Helitrons; DMX: Maverick; DTX: TIRs elements; DXX: Other Class II transposons; RIX: LINEs; RLX: LTR retrotransposons; RSX: SINEs; RXX_TRIM: Terminal Repeat Transposons in Miniature; RPX: Penelopes. (DOCX) [file pone.0257996.s001.docx]

Table S1 Coverage of *M. destructor* chromosomes in TEs

|  | Coverage of  *M. destructor* chromosomes (%) | | | | |
| --- | --- | --- | --- | --- | --- |
| TE order | **A1** | **A2** | **X1** | **X2** | **Un** |
| DHX | 0.0248994 | 0.0346423 | 0.03268208 | 0.03863166 | 0.179905 |
| DMX | 0.2622911 | 0.061172 | 0.1378757 | 0.0222172 | 0.301774 |
| DTX | 2.144545 | 1.531214 | 1.201862 | 1.327318 | 1.94279 |
| DXX | 0.01963797 | 0.00874026 | 0.00266421 | 0.00045192 | 0.0260891 |
| MITEs | 1.908212 | 1.406236 | 0.942653 | 0.779362 | 1.50323 |
| RIX | 0.455782 | 0.813136 | 0.281062 | 0.415326 | 0.676072 |
| RLX | 2.14 | 2.26 | 1.88 | 1.62 | 1.84 |
| RSX | 0.0539145 | 0.0546491 | 0.0770514 | 0.1041238 | 0.0618606 |
| RXX-TRIM | 0.0027059 | 0.01777508 | 0.01223497 | 0.00470528 | 0.0168497 |
| RPX | 0.08 | 0.056 | 0.011 | 0.0025 | 0.13 |
