# Supplementary material for: A Genomic Survey of Mayetiola destructor Mobilome Provides New Insights into the Evolutionary History of Transposable Elements in the Cecidomyiid Midges
Source: PLoS One. 2021 Oct 11;16(10):e0257996. doi: 10.1371/journal.pone.0257996 (PMC8504770; doi:10.1371/journal.pone.0257996)
Supplement: S2 Table — (DOCX) [file pone.0257996.s002.docx]

Table S2 Distribution of TEs superfamilies in the genome of *Mayetiola destructor*

|  |  |  | |  |  | **Consensus representing each lineage (bp)** | | |  |  |
| --- | --- | --- | --- | --- | --- | --- | --- | --- | --- | --- |
| **Class** | **Order** | **Superfamilies** | | **Coverage in bp** | **Number of lineages** | **Minimum size** | **Maximum size** | **Median size** | **Copy number** | **FLC number** |
| **Class I** | LTR | *Ty3/gypsy* | | 745322 | 22 | 356 | 4079 | 834.5 | 1442 | 92 |
|  |  | *Bel-Pao* | | 1023875 | 12 | 680 | 3203 | 1707 | 2208 | 23 |
|  |  | *Ty1/Copia* | | 10805 | 3 | 959 | 4500 | 1500 | 167 | 17 |
|  |  | Autres LTRs | | 9588 | 1 | 894 | 894 | 894 | 21 | 1 |
|  | N-LTR | LINEs | *Jockey* | 356624 | 14 | 510 | 2907 | 1050 | 815 | 32 |
|  |  |  | *I* | 51241 | 4 | 741 | 6950 | 850 | 123 | 21 |
|  |  | Autres LINEs | | 182994 | 10 | 707 | 1230 | 915.5 | 861 | 21 |
|  |  | SINEs | | 52834 | 3 | 520 | 601 | 560.5 | 312 | 4 |
|  | PLEs | *Penelopes* | | 26689 | 2 | 685 | 786 | 887 | 94 | 4 |
|  | Retrotransposons TRIM | | | 12372 | 2 | 354 | 357 | 350 | 45 | 4 |
| **Total Class I** | | | | **2472344** | **73** | **6406** | **25507** | **9548.5** | **6088** | **219** |
| **Class II** | TIR | *Tc1/mariner* | | 347209 | 28 | 535 | 2713 | 928.5 | 956 | 73 |
|  |  | *hAT* | | 325844 | 24 | 534 | 4500 | 1000 | 1150 | 75 |
|  |  | *Mutator* | | 20893 | 2 | 749 | 2301 | 1525 | 28 | 4 |
|  |  | *Harbinger* | | 16440 | 1 | 1900 | 1900 | 1900 | 69 | 2 |
|  |  | *CACTA* | | 29431 | 2 | 1042 | 1664 | 1353 | 60 | 12 |
|  |  | Autres TIRs | | 835474 | 22 | 785 | 1391 | 1090.5 | 2184 | 63 |
|  |  | MITEs | | 1338036 | 84 | 360 | 1736 | 503 | 5.681 | 557 |
|  | Helitron | *Helitron* | | 121020 | 7 | 666 | 2453 | 2006 | 220 | 28 |
|  | Maverick | *Maverick* | | 228968 | 10 | 674 | 4455 | 1243.5 | 408 | 31 |
| **Total Class II** | | | | **3263315** | **181** | **7245** | **23113** | **11549.5** | **5080.681** | **845** |
| noCats | | | | 1775484 | 738 | 169 | 7605 | 792 | 63344 | 1928 |
