# Supplementary material for: A Genomic Survey of Mayetiola destructor Mobilome Provides New Insights into the Evolutionary History of Transposable Elements in the Cecidomyiid Midges
Source: PLoS One. 2021 Oct 11;16(10):e0257996. doi: 10.1371/journal.pone.0257996 (PMC8504770; doi:10.1371/journal.pone.0257996)
Supplement: S6 Fig — The consensuses identified in Mayetiola destructor are marked by triangles. Bootstrap values less than 50% are eliminated. The tree is built by the ML method (model HKY85) with a bootstrap of 1000 repetitions. The clades of mariner-like elements (MLEs), Tc1-like elements (TLEs) and pogo-like elements are highlighted in pink, green and orange, respectively. The three shades in the MLEs clade refer to the mauritiana, irritans and rosa subfamilies from the top to the bottom. (DOCX) [file pone.0257996.s009.docx]

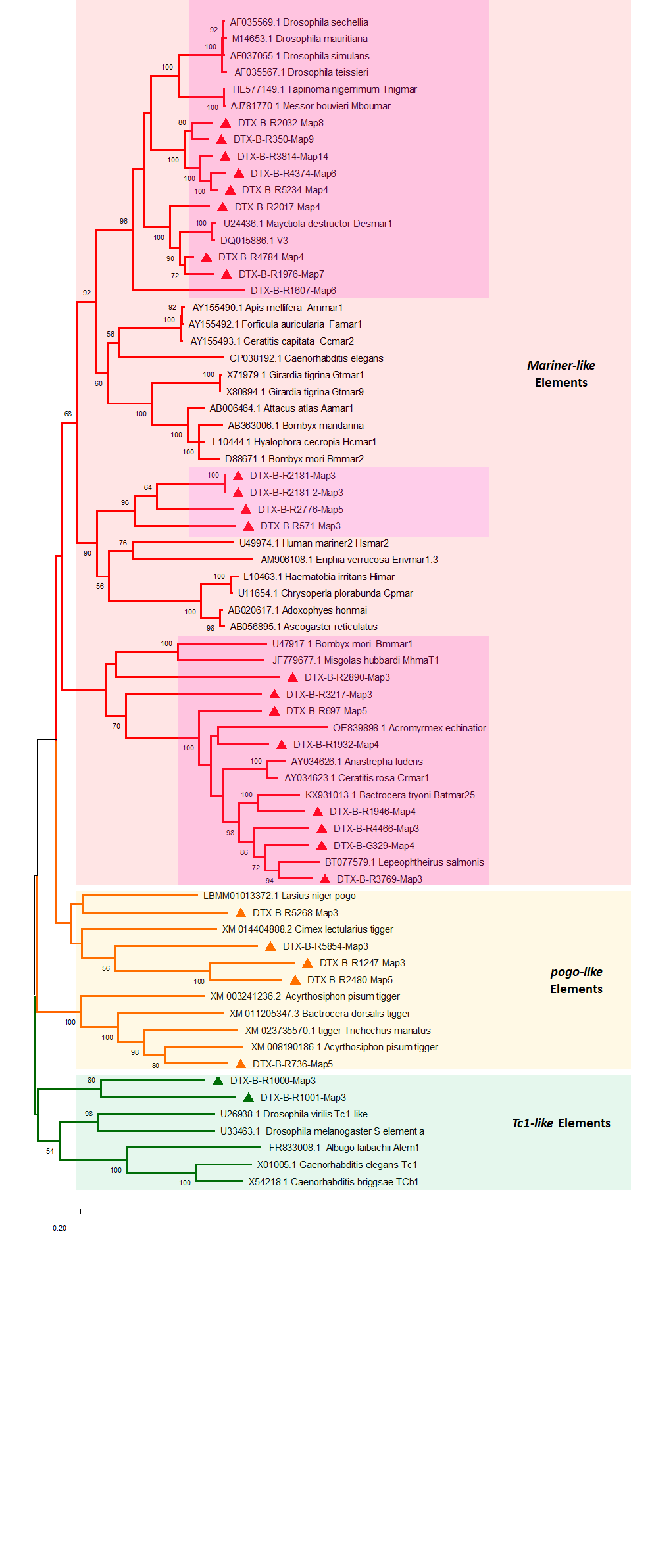


**S6 Fig.** Classification of the 28 consensus Tc1 / mariner superfamily of *Mayetiola destructor*

The consensuses identified in *Mayetiola destructor* are marked by triangles. Bootstrap values ​​less than 50% are eliminated. The tree is built by the ML method (model HKY85) with a bootstrap of 1000 repetitions.
