# Supplementary material for: A Genomic Survey of Mayetiola destructor Mobilome Provides New Insights into the Evolutionary History of Transposable Elements in the Cecidomyiid Midges
Source: PLoS One. 2021 Oct 11;16(10):e0257996. doi: 10.1371/journal.pone.0257996 (PMC8504770; doi:10.1371/journal.pone.0257996)
Supplement: S8 Fig — The consensuses identified in Mayetiola destructor are marked by triangles. Bootstrap values less than 50% are eliminated. The tree is built by the ML method (model HKY85) with a bootstrap of 1000 repetitions. (DOCX) [file pone.0257996.s011.docx]

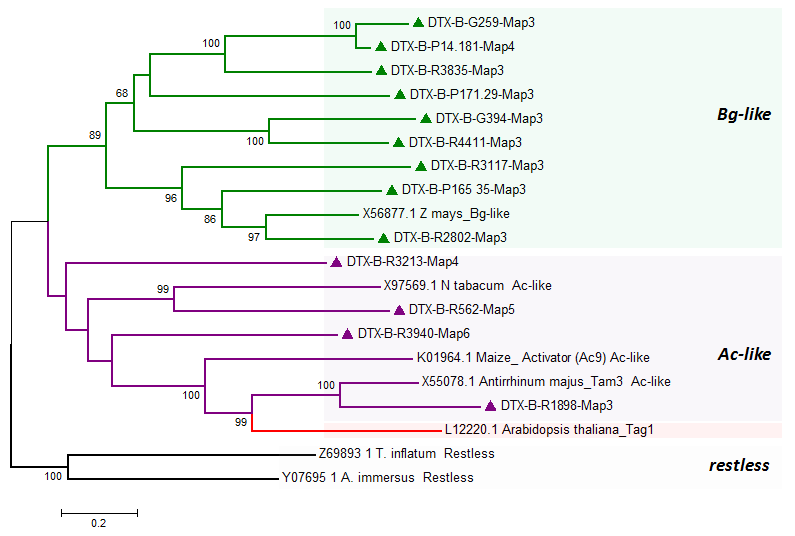


**S8 Fig.** Classification of *Mayetiola destructor* *hAT* superfamily elements within isolated groups in plants and fungi

The consensuses identified in *Mayetiola destructor* are marked by triangles. Bootstrap values ​​less than 50% are eliminated. The tree is built by the ML method (model HKY85) with a bootstrap of 1000 repetitions.
