# Supplementary material for: A Genomic Survey of Mayetiola destructor Mobilome Provides New Insights into the Evolutionary History of Transposable Elements in the Cecidomyiid Midges
Source: PLoS One. 2021 Oct 11;16(10):e0257996. doi: 10.1371/journal.pone.0257996 (PMC8504770; doi:10.1371/journal.pone.0257996)
Supplement: S9 Fig — The consensuses identified in Mayetiola destructor are marked by triangles. Bootstrap values less than 50% are eliminated. The tree is built by the ML method (model HKY85) with a bootstrap of 1000 repetitions. The reference sequences are isolated from the following species: AG: Anopheles gambiae; DF: Drosophila ficusphila; DBP: Drosophila bipectinata; LMi: Locusta migratoria; HRD: Drosophila rhopaloa; CMi: Callorhinchusmilii; SPur: Strongylocentrotus purpuratus; SP: Strongy locentrotus purpuratus; OS: Oryza sativa. (DOCX) [file pone.0257996.s012.docx]

**S9 Fig.** Phylogeny of the seven consensus helitrons of *Mayetiola destructor*

The consensuses identified in *Mayetiola destructor* are marked by triangles. Bootstrap values ​​less than 50% are eliminated. The tree is built by the ML method (model HKY85) with a bootstrap of 1000 repetitions. The reference sequences are isolated from the following species: AG: *Anopheles gambiae*; DF: *Drosophila ficusphila*; DBP: *Drosophila bipectinata*; LMi: *Locusta migratoria*; HRD: *Drosophila rhopaloa*; CMi: *Callorhinchus milii*; SPur: *Strongylocentrotus purpuratus*; SP: *Strongylocentrotus purpuratus*; OS: *Oryza sativa*
