# Supplementary material for: A Genomic Survey of Mayetiola destructor Mobilome Provides New Insights into the Evolutionary History of Transposable Elements in the Cecidomyiid Midges
Source: PLoS One. 2021 Oct 11;16(10):e0257996. doi: 10.1371/journal.pone.0257996 (PMC8504770; doi:10.1371/journal.pone.0257996)
Supplement: S10 Fig — The consensuses identified in Mayetiola destructor are marked by triangles. Bootstrap values less than 50% are eliminated. The tree is built by the ML method (model HKY85) with a bootstrap of 1000 repetitions. The reference sequences are isolated from the following species: DR: Danio rerio; DBP: Drosophila bipectinata; NV: Nematostella vectensis; NVi: Nasonia vitripennis; TC: Tribolium castaneum; DEu: Drosophila eugracilis; DK: Drosophila kikkawai. (DOCX) [file pone.0257996.s013.docx]

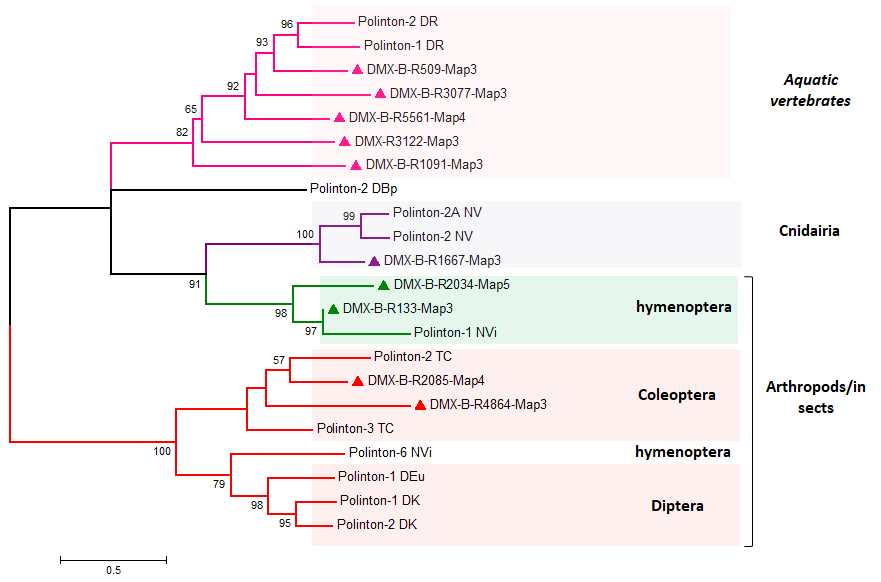


**S10 Fig.** Phylogeny of Maverick / Polintons elements

The consensuses identified in *Mayetiola destructor* are marked by triangles. Bootstrap values ​​less than 50% are eliminated. The tree is built by the ML method (model HKY85) with a bootstrap of 1000 repetitions. The reference sequences are isolated from the following species: DR: *Danio rerio*; DBP: *Drosophila bipectinata*; NV: *Nematostella vectensis*; NVi: *Nasonia vitripennis*; TC: *Tribolium castaneum*; DEu: *Drosophila eugracilis*; DK: *Drosophila kikkawai*
